# Supplementary material for: Genetic correlations and genome-wide associations of cortical structure in general population samples of 22,824 adults
Source: Nat Commun. 2020 Sep 22;11:4796. doi: 10.1038/s41467-020-18367-y (PMC7508833; doi:10.1038/s41467-020-18367-y)
Supplement: Supplementary file 1 — Supplementary Information [file 41467_2020_18367_MOESM1_ESM.pdf]

Genetic correlations and genome-wide associations of cortical structure in general population samples of 22,824 adults

Hofer, Roshchupkin, Adams et al.

Supplementary Information

Supplementary Methods

Cohorts

Quality control

Freesurfer defined cortical regions

Supplementary Figures

Supplementary Figure 1. Regional association plots for global cortical surface area.

Supplementary Figure 2. Regional association plots for global cortical volume.

Supplementary Figure 3. Regional association plots for postcentral cortical thickness, surface area and volume.

Supplementary Figure 4. QQ plots of global cortical thickness, surface area and volume meta-analyses.

Supplementary Figure 5. QQ plots of regional cortical thickness meta-analyses.

Supplementary Figure 6. QQ plots of regional cortical surface area meta-analyses.

Supplementary Figure 7. QQ plots of regional cortical volume meta-analyses.

Supplementary Figure 8. Pathway analysis of 44 genes mapped to independent lead SNPs of cortical thickness.

Supplementary Figure 9. Pathway analysis of 105 genes mapped to independent lead SNPs of cortical surface area.

Supplementary Figure 10. Pathway analysis of 82 genes mapped to independent lead SNPs of cortical volume.

Supplementary Figure 11. Regional heritability estimates based on common SNPs.

Supplementary Figure 12. Genetic correlation between cortical thickness, surface area and volume within cortical regions.

Supplementary Figure 13. Genetic correlation between regional cortical thickness.

Supplementary Figure 14. Genetic correlation between regional cortical surface area.

Supplementary Figure 15. Genetic correlation between regional cortical volume.

Supplementary Figure 16. Genetic correlation between cortical thickness and other GWAS phenotypes.

Supplementary Figure 17. Genetic correlation between cortical surface area and other GWAS phenotypes.

Supplementary Figure 18. Genetic correlation between cortical volume and other GWAS phenotypes.

Supplementary Notes

Supplementary Note 1. Acknowledgments

Supplementary References

Supplementary Methods

Cohorts

Atherosclerosis Risk in Communities Study (ARIC)

The ARIC study is a population-based cohort study of atherosclerosis and clinical atherosclerotic diseases[1]. At its inception (1987-1989), 15,792 men and women, including 11,478 white and 4,266 black participants were recruited from four U.S. communities: Suburban Minneapolis, Minnesota; Washington County, Maryland; Forsyth County, North Carolina; and Jackson, Mississippi. In the first 3 communities, the sample reflects the demographic composition of the community. In Jackson, only black residents were enrolled. Participants were between age 45 and 64 years at their baseline examination in 1987-1989 when blood was drawn for DNA extraction and participants consented to genetic testing. Vascular risk factors and outcomes, including transient ischemic attack, stroke and dementia, were determined in a standard fashion. During the first 2 years (1993-1994) of the third ARIC examination (V3), participants aged 55 and older from the Forsyth County and Jackson sites were invited to undergo cranial MRI. This subgroup of individuals with MRI scanning represents a random sample of the full cohort because examination dates were allocated at baseline through randomly selected induction cycles.

Austrian Stroke Prevention Study (ASPS)

The ASPS study is a single center prospective follow-up study on the effects of vascular risk factors on brain structure and function in the normal elderly population of the city of Graz, Austria. The procedure of recruitment and diagnostic work-up of study participants has been described previously[2,3]. A total of 2007 participants were randomly selected from the official community register stratified by gender and 5 year age groups. Individuals were excluded from the study if they had a history of neuropsychiatric disease, including previous stroke, transient ischemic attacks, and dementia, or an abnormal neurologic examination determined on the basis of a structured clinical interview and a physical and neurologic examination. During 2 study periods between September 1991 and March 1994 and between January 1999 and December 2003 an extended diagnostic work-up including MRI and neuropsychological testing was done in 1076 individuals aged 45 to 85 years randomly selected from the entire cohort: 509 from the first period and 567 from the second. In 1992, blood was drawn from all study participants for DNA extraction. They were all European Caucasians. Genotyping was performed in 996 participants, and the 182 who underwent MRI scanning at baseline were available for these analyses.

Austrian Stroke Prevention Family Study (ASPS-Fam)

ASPS-Fam is a prospective single-center community-based study on the cerebral effects of vascular risk factors in the normal aged population of the city of Graz, Austria[4,5]. ASPS-Fam represents an extension of the Austrian Stroke Prevention Study (ASPS), which was established in 1991[2,3]. Between 2006 and 2013, study participants of the ASPS and their first-grade relatives were invited to enter ASPS-Fam. Inclusion criteria were no history of previous stroke

or dementia and a normal neurologic examination. A total of 419 individuals from 176 families were included into the study. The number of members per family ranged from 2 to 6. The entire cohort underwent a thorough diagnostic workup including clinical history, laboratory evaluation, cognitive testing, and an extended vascular risk factor assessment. They were all European Caucasians. Those 297 participants who passed genotyping quality control and underwent MRI scanning were available for these analyses.

Cardiovascular Health Study (CHS)

The CHS is a population-based cohort study of risk factors for coronary heart disease and stroke in adults ≥ 65 years conducted across four field centers[6]. The original predominantly European ancestry cohort of 5,201 persons was recruited in 1989-1990 from random samples of the Medicare eligibility lists; subsequently, an additional predominantly African-American cohort of 687 persons was enrolled for a total sample of 5,888. Blood samples were drawn from all participants at their baseline examination and DNA was subsequently extracted from available samples. Genotyping was performed at the General Clinical Research Center's Phenotyping/Genotyping Laboratory at Cedars-Sinai among CHS participants who consented to genetic testing and had DNA. European ancestry participants were excluded from the GWAS study sample due to the presence at study baseline of coronary heart disease, congestive heart failure, peripheral vascular disease, valvular heart disease, stroke or transient ischemic attack or lack of available DNA. Among those with successful GWAS, 567 European ancestry participants had available FreeSurfer measures for this analysis. CHS was approved by institutional review committees at each field center and individuals in the present analysis had available DNA and gave informed consent including consent to use of genetic information for the study of cardiovascular disease.

Erasmus Rucphen Family Study (ERF)

The Erasmus Rucphen Family genetic isolate study (ERF) is a prospective family based study located in Southwest of the Netherlands[7,8]. This young genetic isolate was founded in the mid-eighteenth century and minimal immigration and marriages occurred between surrounding settlements due to social and religious reasons. The ERF study population includes 3465 individuals that are living descendants of 22 couples with at least six children baptized. Informed consent has been obtained from patients where appropriate. The study protocol was approved by the medical ethics board of the Erasmus Medical Center Rotterdam, the Netherlands.

Framingham Heart Study (FHS)

The FHS is a three-generation, single-site, community-based, ongoing cohort study that was initiated in 1948 to investigate the risk factors for cardiovascular disease. It now comprises 3 generations of participants: the Original cohort followed since 1948[9]; their Offspring and spouses of the Offspring (Gen 2), followed since 1971[10]; and children from the largest Offspring families enrolled in 2000 (Gen 3)[11]. The Original cohort enrolled 5,209 men and women who comprised two-thirds of the adult population then residing in Framingham, MA. Survivors continue to receive biennial examinations. The Offspring cohort comprises 5,124 persons (including 3,514 biological offspring)

who have been examined approximately once every 4 years. The Third-generation includes 4,095 participants with at least one parent in the Offspring Cohort. The first two generations were invited to undergo an initial brain MRI in 1999-2005, and for Gen 3, brain MRI began in 2009. The population of Framingham was virtually entirely white (Europeans of English, Scots, Irish and Italian descent) in 1948 when the Original cohort was recruited. Self-reports of ethnicity across all three generations were 99.7% whites, reflecting the ethnicity of the population of Framingham in 1948. FHS participants had DNA extracted and provided consent for genotyping, and eligible participants underwent genome-wide genotyping.

Lothian Birth Cohort 1936 (LBC1936)

The LBC1936 consists of relatively healthy individuals assessed on cognitive and medical measures at age 70 years (n=1,091), and again with brain imaging traits at 73 years of age (n=866). They were born in 1936, most took part in the Scottish Mental Survey of 1947, and almost all lived independently in the Lothian region of Scotland. A full description of participant recruitment and testing can be found elsewhere[12-14]. The study was approved by the Lothian (REC 07/MRE00/58) and Scottish Multicentre (MREC/01/0/56) Research Ethics Committees and all subjects give written informed consent.

LIFE-Adult

LIFE-Adult is a population-based study and a part of the large-scale research project LIFE (Leipzig Research Center for Civilization Diseases). 10,000 residents (main age range 40 – 79) from the district of Leipzig (Saxony, Germany) were recruited and extensively phenotyped for a number of disease and environmental parameters (see Loeffler et al.[15] for a description of the assessment programme). All subjects gave written informed consent to participate in the study. The procedures were conducted according to the Declaration of Helsinki and approved by the University of Leipzig's ethics committee (registration-number: 263-2009-14122009).

Sydney Memory and Ageing Study (MAS)

The Sydney Memory and Ageing Study recruited participants aged 70-90 years of age randomly from the community in Sydney, Australia (N=1037 at baseline). Exclusion criteria included a diagnosis of dementia, schizophrenia or a progressive malignancy. Comprehensive data was collected, including an extensive cognitive battery, demographics and medical history. Most participants provided a blood sample for genetic and biochemistry analyses. A subset of participants also underwent neuroimaging. Ethics approval for the study was provided by the University of New South Wales and the Illawarra Area Health Service Human Research Ethics Committees. All participants gave written informed consent. More information is provided in Sachdev et al. 2010[16].

Older Australian Twin Study (OATS)

Twins aged 65 years and over were recruited from Twins Registry Australia and through a recruitment drive from the three eastern Australian states into the Older Australian Twins Study (N=623 at baseline). Exclusion criteria included insufficient English to finish the assessment. A comprehensive assessment was undertaken including an extensive cognitive battery and blood was collected for genetic and biochemistry analyses. For a subset, neuroimaging was also performed. Ethics approval for the study was provided by Twins Registry Australia, University of New South Wales, University of Melbourne, Queensland Institute of Medical Research and the South Eastern Sydney and Illawarra Area Health Service University of New South Wales and the Illawarra Area Health Service Human Research Ethics Committees. All participants gave written informed consent. More information is provided in Sachdev et al. 2009[17].

Rotterdam Study (RSI, RSII, RSIII)

The Rotterdam Study is a prospective, population-based cohort study among individuals living in the well-defined Ommoord district in the city of Rotterdam in The Netherlands[18]. The aim of the study is to determine the occurrence of cardiovascular, neurological, ophthalmic, endocrine, hepatic, respiratory, and psychiatric diseases in elderly people. The cohort was initially defined in 1990 among approximately 7,900 persons, aged 55 years and older, who underwent a home interview and extensive physical examination at the baseline and during follow-up rounds every 3-4 years (RS-I). The cohort was extended in 2000/2001 (RS-II, 3,011 individuals aged 55 years and older) and 2006/2008 (RS-III, 3,932 subjects, aged 45 and older). Written informed consent was obtained from all participants and the Medical Ethics Committee of the Erasmus Medical Center, Rotterdam, approved the study.

Study of Health in Pomerania (SHIP)

The Study of Health in Pomerania (SHIP) is a population-based project in West Pomerania, the north-east area of Germany.[19] A sample from the population aged 20 to 79 years was drawn from population registries. First, the three cities of the region (with 17,076 to 65,977 inhabitants) and the 12 towns (with 1,516 to 3,044 inhabitants) were selected, and then 17 out of 97 smaller towns (with less than 1,500 inhabitants), were drawn at random. Second, from each of the selected communities, subjects were drawn at random, proportional to the population size of each community and stratified by age and gender. Only individuals with German citizenship and main residency in the study area were included. Finally, 7,008 subjects were sampled, with 292 persons of each gender in each of the twelve five-year age strata. In order to minimize drop-outs by migration or death, subjects were selected in two waves. The net sample (without migrated or deceased persons) comprised 6,267 eligible subjects. Selected persons received a maximum of three written invitations. In case of non-response, letters were followed by a phone call or by home visits if contact by phone was not possible. The SHIP population finally comprised 4,308 participants (corresponding to a final response of 68.8%).

Study of Health in Pomerania Trend (SHIP-Trend)

The Study of Health in Pomerania (SHIP-Trend) is a population-based cohort study in West Pomerania, a region in the northeast of Germany, assessing the prevalence and incidence of common population-relevant diseases and their risk factors.[19] Baseline examinations for SHIP-Trend were carried out between 2008 and 2012, comprising 4,420 participants aged 20 to 81 years. Study design and sampling methods were previously described. The medical ethics committee of the University of Greifswald approved the study protocol, and oral and written informed consents were obtained from each of the study participants.

Saguenay Youth Study (SYS)

The Saguenay Youth Study (SYS) is a two-generational study of adolescents and their parents (n=1,029 adolescents and 962 parents) aimed at investigating the etiology, early stages and trans-generational trajectories of common cardio-metabolic and brain diseases. The cohort was recruited from the genetic founder population of the Saguenay Lac St. Jean region of Quebec, Canada. The participants underwent extensive (15-hour) phenotyping, including an hour-long recording of beat-by-beat blood pressure, magnetic resonance imaging of the brain and abdomen, and serum lipidomic profiling with LC-ESI-MS. All participants have been genome-wide genotyped (with ~8M imputed SNPs) and a subset of them (144 adolescents and their 288 parents) has been genome-wide epityped (whole blood DNA, Infinium HumanMethylation450K BeadChip). These assessments are complemented by a detailed evaluation of each participant in a number of domains, including cognition, mental health and substance use, diet, physical activity and sleep, and family environment. The data collection took place during 2003-2012 in adolescents (full) and their parents (partial), and during 2012-2015 in parents (full). The Research Ethics Committee of the Chicoutimi Hospital approved the study protocol. Written informed consent was also obtained from all the participants.

Three-City Dijon (3C-Dijon)

The 3C study is conducted in three French cities (Bordeaux, Dijon, and Montpellier), comprising 9,294 participants, designed to estimate the risk of dementia and cognitive impairment attributable to vascular factors.1 Eligibility criteria included living in the city and being registered on the electoral rolls in 1999, 65 years or older, and not institutionalized. The 3C-Dijon study recruited 4,931 individuals. The overall design of the 3C-Dijon study is detailed elsewhere[20-22]. Participants aged less than 80 years and enrolled between June 1999 and September 2000 (n=2,763) were invited to undergo a brain MRI. Although 2,285 subjects agreed to participate (82.7%), because of financial limitations, 1,924 MRI scans were performed. DNA samples of 3C-Dijon participants were genotyped at the Centre National de Génotypage, Evry, France, with Illumina Human610IQuad® BeadChips.

United Kingdom Biobank (UKBB)

The UKBB is a large-scale epidemiological study of over 500,000 individuals aged 40-69 years from the United

Kingdom (http://www.ukbiobank.ac.uk). The analyses presented here use data that were accessed via application 1155. Genetic data are available for the majority of these individuals[23] and as of 15 July 2017 13,269 of these participants had participated in a multimodal imaging sub-study [24,25]. The analyses presented here use the cortical measurements of the 8.213 participants released by the UKBB which are derived from the T1 Brain MRI, the extraction of these measures using FreeSurfer software version 6.0. These data were not visually QCed (as the required files were not available for download). However, we removed outliers by setting data points more than 3 standard deviations from the mean to missing. The genetic data used for these analyses uses only those variants imputed using the HRC reference panel. Imputation accuracy and allele frequency were recalculated in the subset of participants with imaging from the raw imputed data using HASE software, quality control filters used in the meta-analyses were applied to the UKBB data prior to analysis. To account for ethnicity, we included only subjects with white British ancestry (base on provided by UK Biobank information). To avoid correct cryptic relationship we excluded all subject with >=3rd degree of genetic relationship.

Vietnam Era Twin Study of Aging (VETSA)

Wave 1 of the longitudinal VETSA project comprised middle-aged male twins aged 51-60 years randomly recruited form the population-based Harvard Drug Study (HDS)[1] between 2003 and 2007[2,3]. Eligibility for the HDS was not based on drug use or any clinical or diagnostic characteristic. VETSA participants were concordant for US military service at some time between 1965 and 1975. Nearly 80% reported no combat experience. The sample is 88.3% non-Hispanic white, 5.3% African-American, 3.4% Hispanic, and 3.0% “other” participants. Based on data from the US National Center for Health Statistics, the sample is very similar to American men in their age range with respect to health and lifestyle characteristics[4]. A total of 445 individuals who had genotyping and analyzable MRI data, and were of European ancestry were included in the present analysis. Once generated, the cortical surface model was manually reviewed and edited for technical accuracy. Minimal manual editing was performed by applying standard, objective editing rules. DNA samples were genotyped at deCODE Genetics (Reykjavík, Iceland) with Illumina HumanOmniExpress-24 v1.0A beadchips.

Enhancing Neuroimaging Genetics through Meta-analysis (ENIGMA)

The ENIGMA sample includes 22,633 individuals of European ancestry from 44 cohorts. The average age was 39.9 (ranging from 3.4 to 91.4) years. The cohorts were either population-based (23 cohorts) or case-control (21 cohorts), where the cases had a psychiatric or neurological disorder. The cohorts included were: 1000BRAINS, ADNI1, ADNI2GO, BETULA, BIG-Affy, BIG-PsychChip, BONN, BrainScale, CARDIFF, CLING, DNS-V3, DNS-V4, FBIRN, FOR2107, GSP, HUBIN, HUNT, IMAGEN, IMpACT, LBC1936, LIBD, MCIC, MPIP, MPRC, MÜNSTER, NCNG, NESDA, NeuroIMAGE, NTR, OATS, PAFIP, PDNZ, PING, PPMI, QTIM, SHIP, SHIP-Trend, Sydney MAS, SYS, TOP, TOP3T, UiO2016, UiO2017, and UMCU.

Quality Control (QC)

Pre-meta-analyis QC

We used the protocol implemented in EasyQC[30] to perform quality control before meta-analysis. SNPs were filtered out based on the following QC criteria:

- missing information (alleles, p-value, effect size, standard error of effect-size, frequency of effect allele, sample size for the SNP, imputation quality)
- impossible values (p-value<0 or p-value>1, standard error of effect-size <=0 or standard error of effect-size = infinity, frequency of effect allele <0 or frequency of effect allele > 1, imputation quality<0)
- non A/C/G/T/I/D markers
- duplicated SNPs (the SNP with the lower sample size was removed)
- monomorphic SNPs
- allele mismatches between input and reference (e.g. A/T in input, A/G in reference)
- allele frequency outliers: remove SNPs where |Freq-Freq.ref|>0.2
- number of individuals in the cohort with this SNP <100
- minor allele count<= 6
- imputation quality < 0.4

Post-meta-analyis QC

After the meta-analysis we removed SNPs with a minor allele frequency (MAF) < 0.05 and which were available in less than 10000 individuals.

Freesurfer defined cortical regions (https://surfer.nmr.mgh.harvard.edu/fswiki/CorticalParcellation)

**Frontal**
Superior Frontal
Rostral Middle Frontal
Caudal Middle Frontal
Pars Opercularis
Pars Triangularis
Pars Orbitalis
Lateral Orbitofrontal
Medial Orbitofrontal
Precentral
Paracentral
Frontal Pole

**Temporal**
Superior Temporal
Middle Temporal
Inferior Temporal
Banks of the Superior Temporal Sulcus
Fusiform
Transverse Temporal
Entorhinal
Temporal Pole
Parahippocampal

**Parietal**
Superior Parietal
Inferior Parietal
Supramarginal
Postcentral
Precuneus

**Occipital**
Lateral Occipital
Lingual
Cuneus
Pericalcarine

**Cingulate**
Rostral Anterior
Caudal Anterior
Posterior
Isthmus

Insula

Supplementary Figures

Supplementary Figure 1. Regional association plots for global cortical surface area.

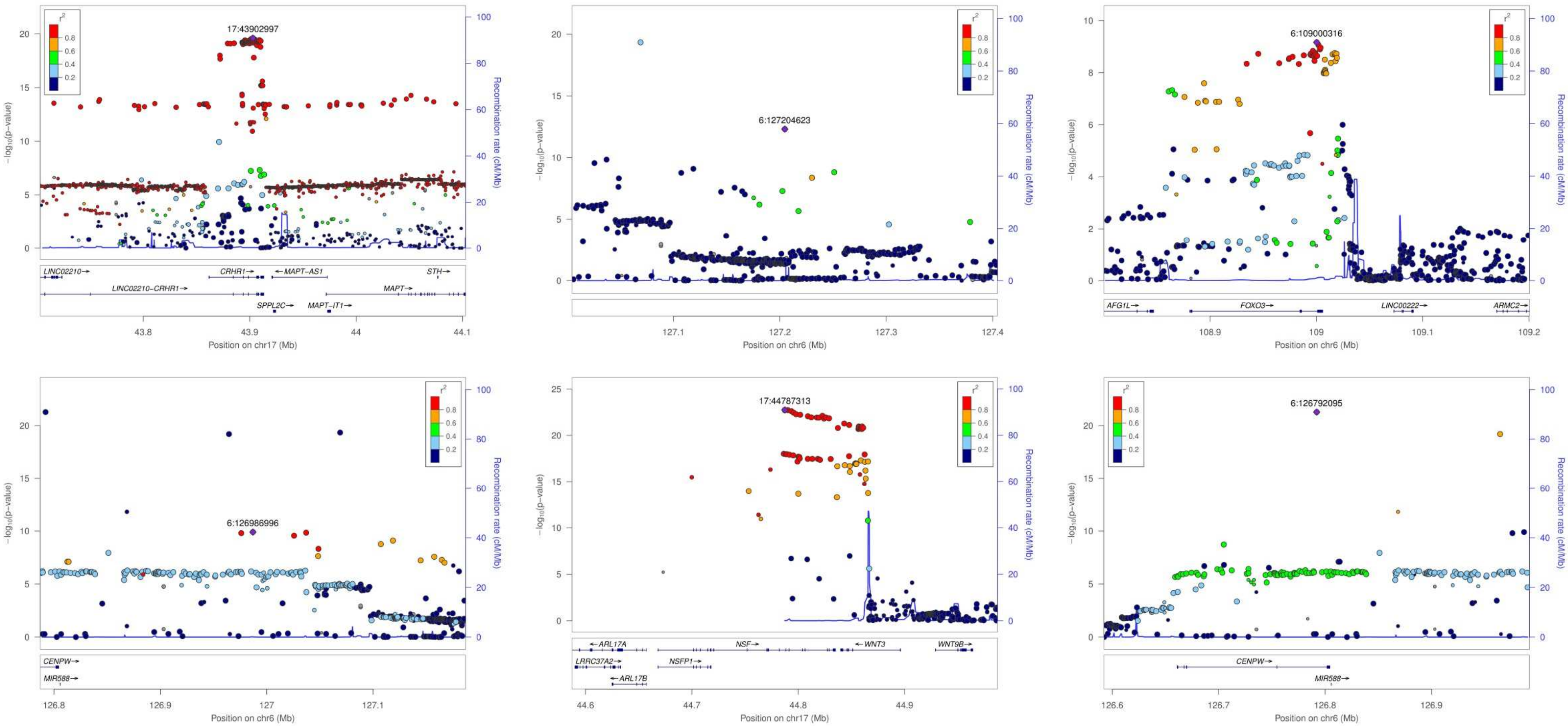

Supplementary Figure 2. Regional association plots for global cortical volume.

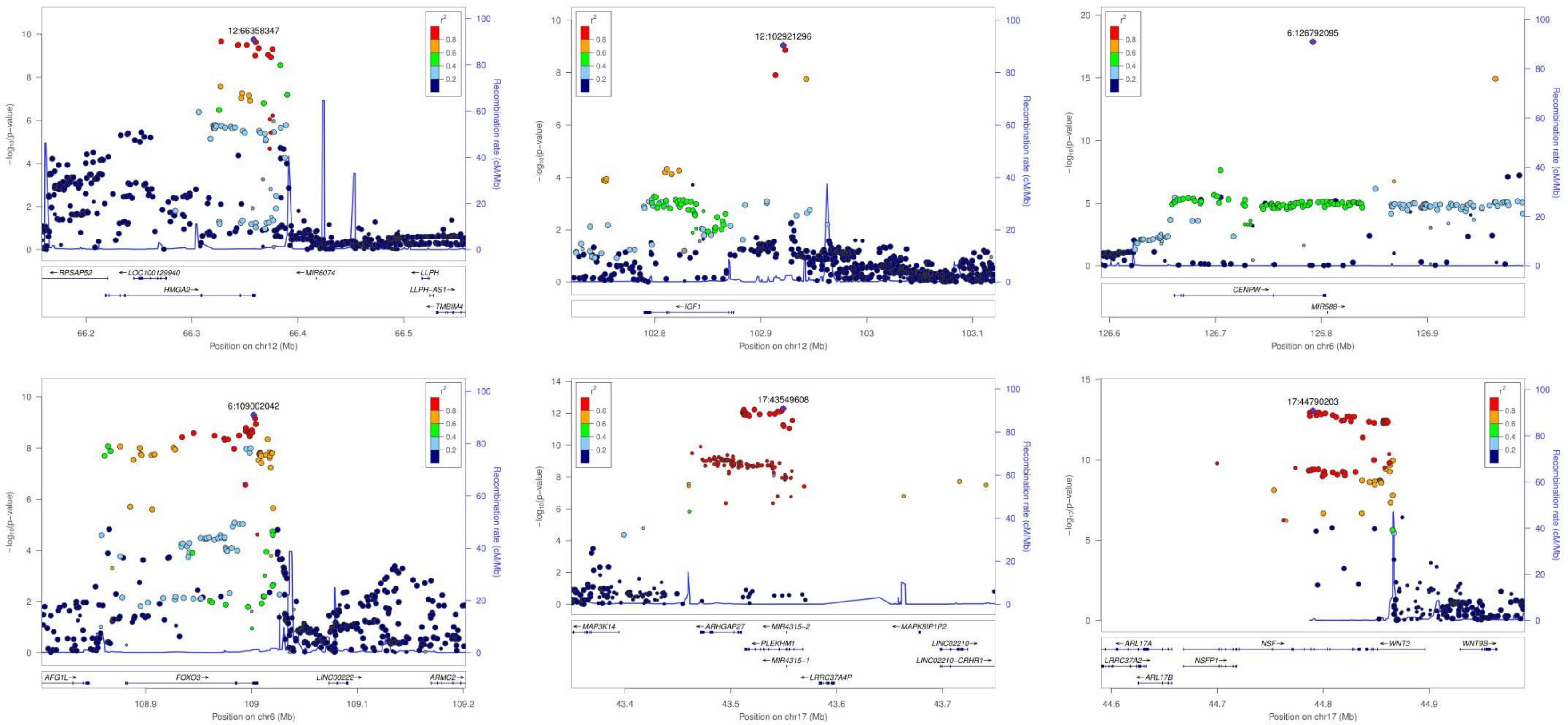

Supplementary Figure 3. Regional association plots of postcentral cortical thickness (CTh), surface area (CSA) and volume (CV).

CTh

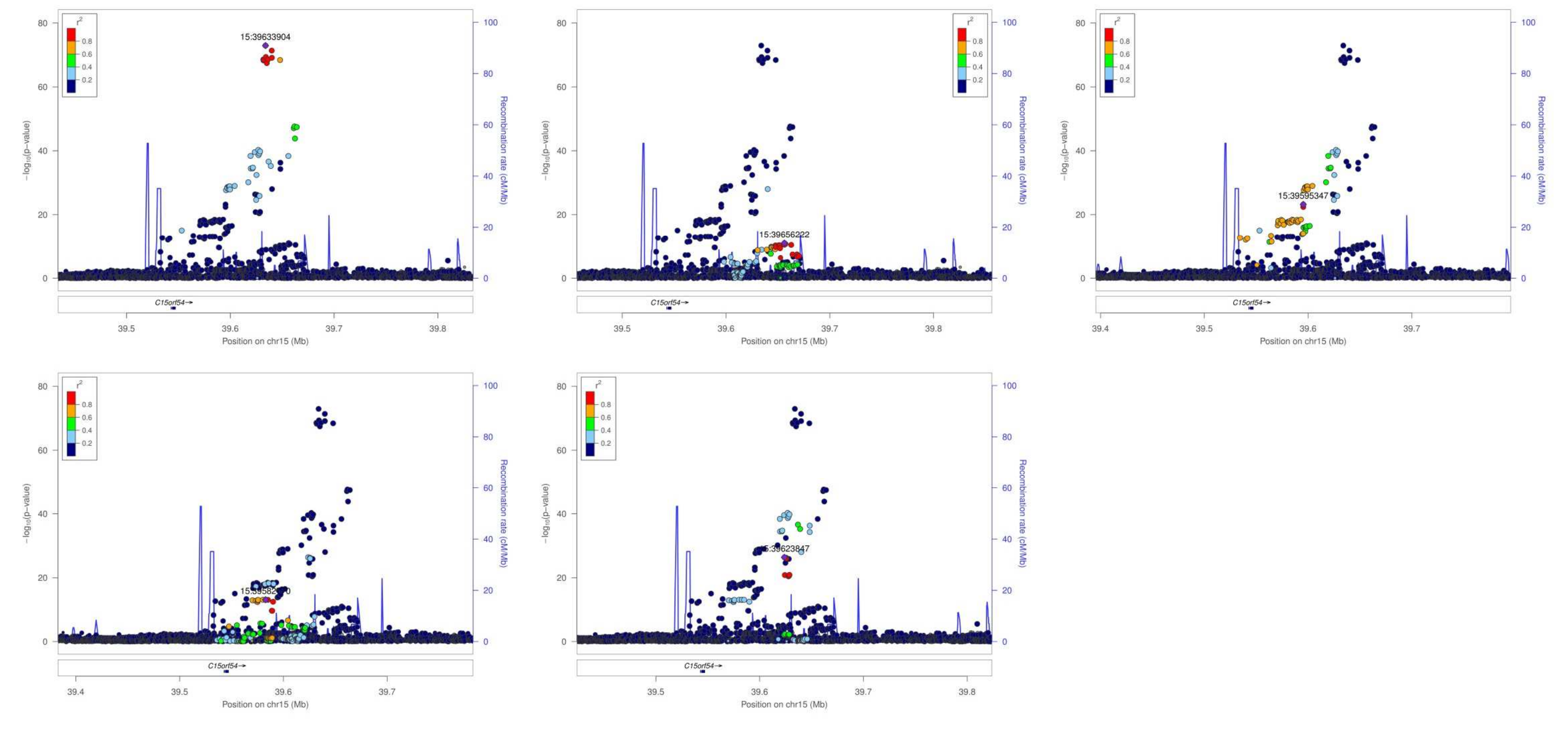

CSA

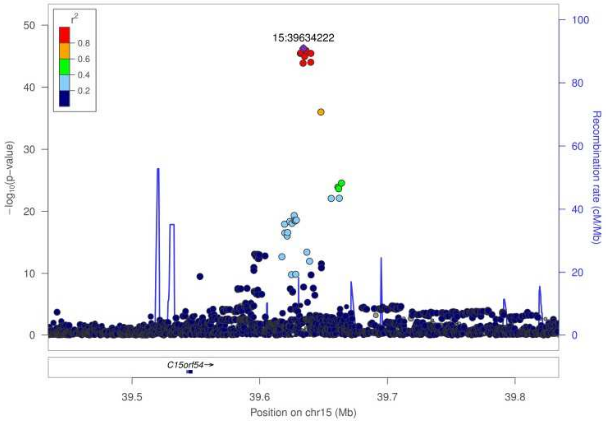
15:39634222
−log10(p−value)
Recombination rate (cM/Mb)
C15orf54→
Position on chr15 (Mb)

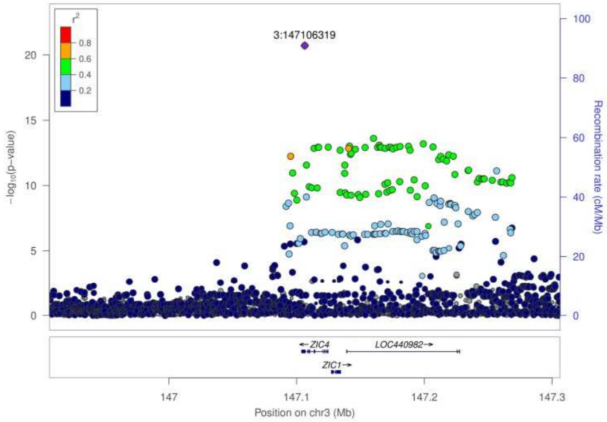
3:147106319
−log10(p−value)
Recombination rate (cM/Mb)
←ZIC4
LOC440982→
ZIC1→
Position on chr3 (Mb)

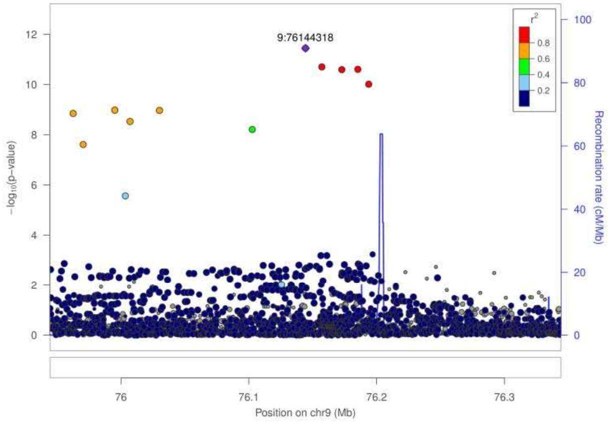
9:76144318
−log10(p−value)
Recombination rate (cM/Mb)
Position on chr9 (Mb)

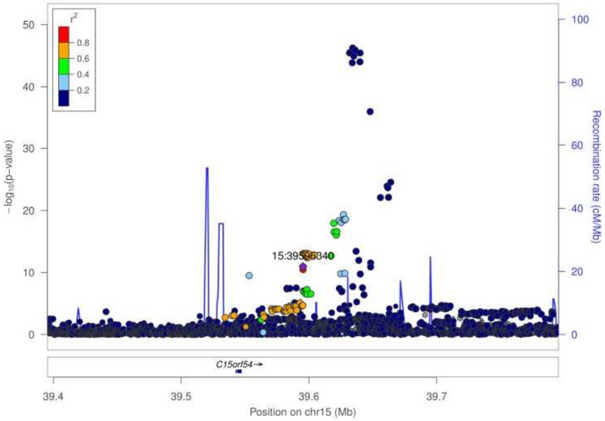
−log10(p−value)
Recombination rate (cM/Mb)
C15orf54→
Position on chr15 (Mb)

CV

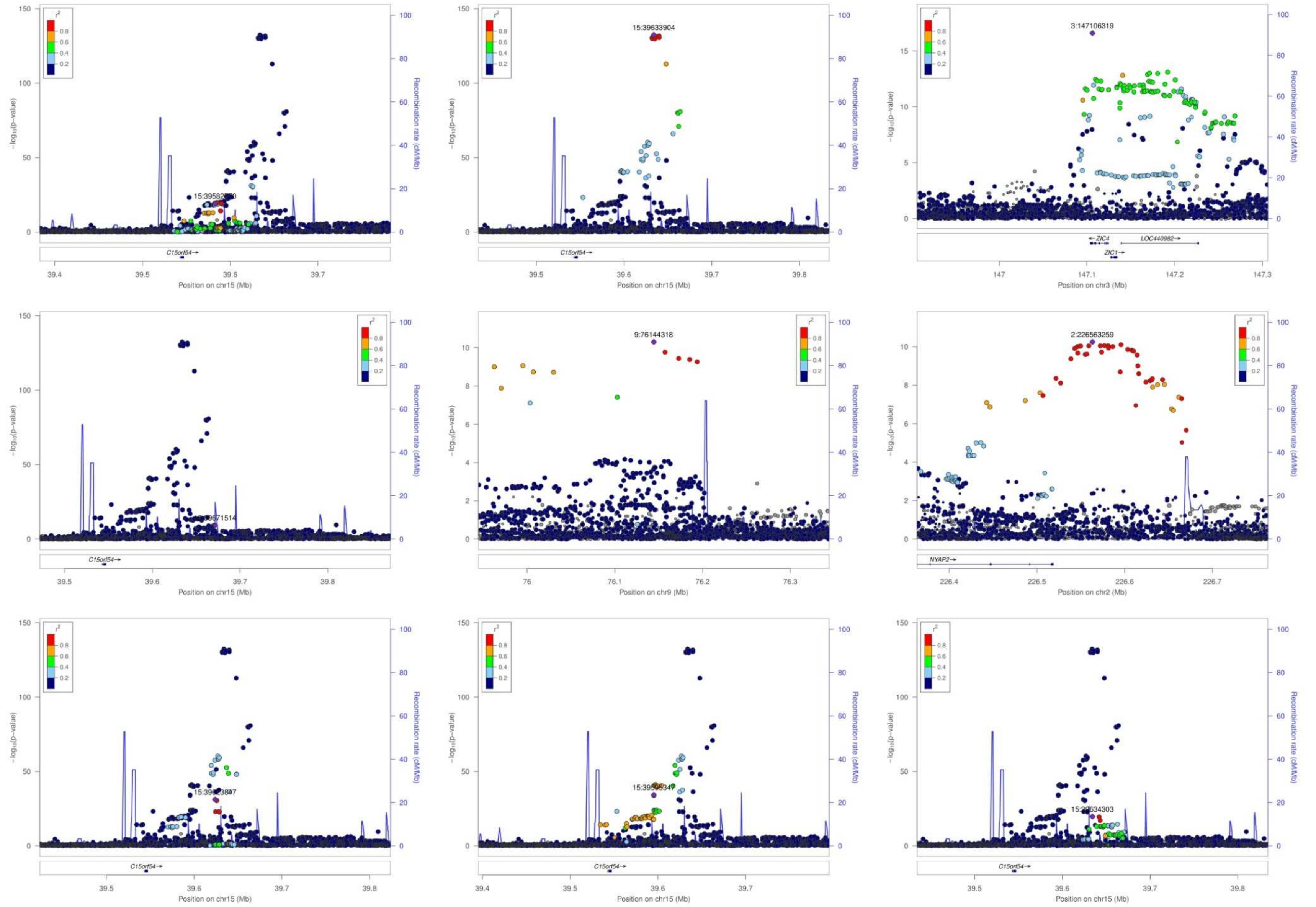

15:39633904
3:147106319
9:76144318
2:226563259
C15orf54
ZIC4
LOC440982
ZIC1
NYAP2
Position on chr15 (Mb)
Position on chr3 (Mb)
Position on chr9 (Mb)
Position on chr2 (Mb)
Recombination rate (cM/Mb)

Supplementary Figure 4. QQ plots of global cortical thickness (CTh), surface area (CSA) and volume (CV) meta-analyses.

**global CTh**	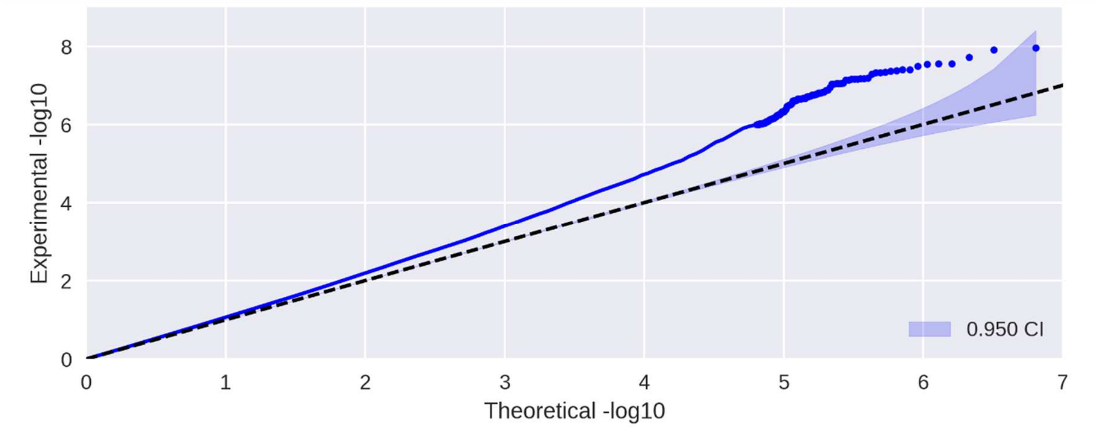
**global CSA**	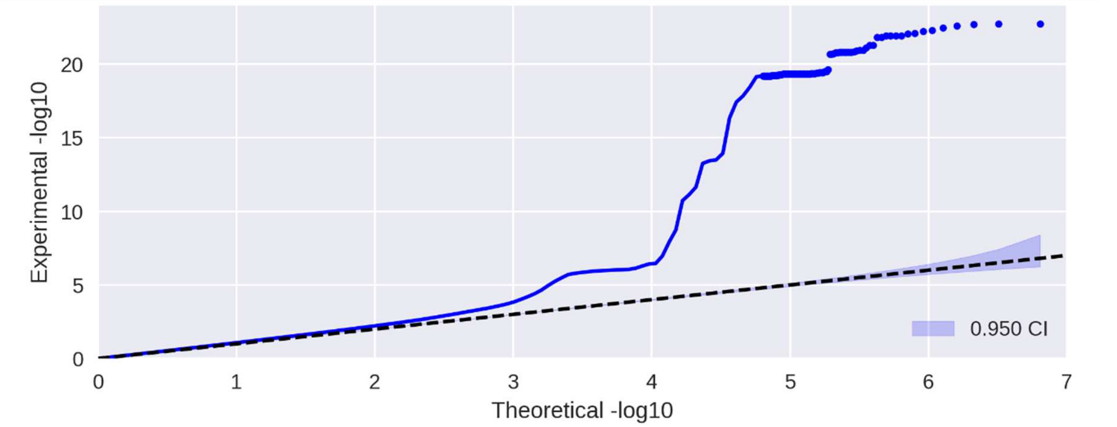
**global CV**	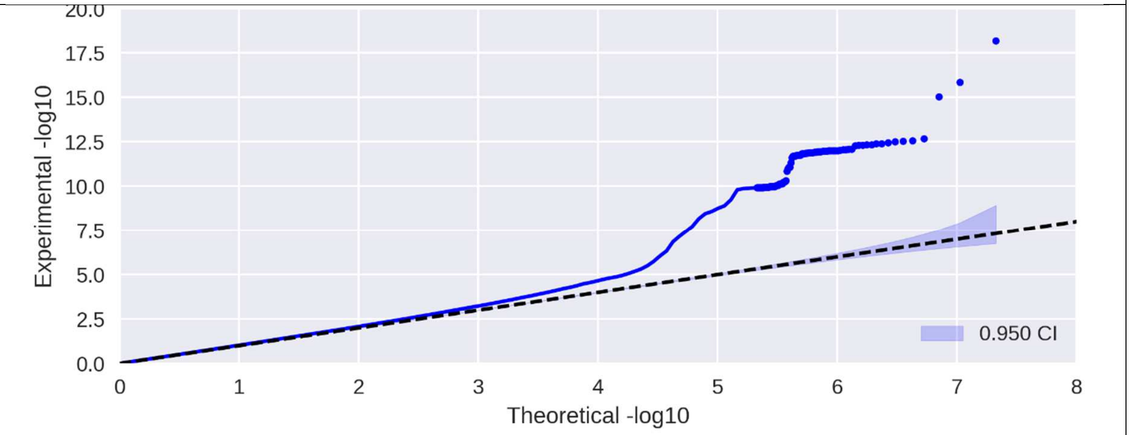

Supplementary Figure 5. QQ plots of regional cortical thickness meta-analyses.

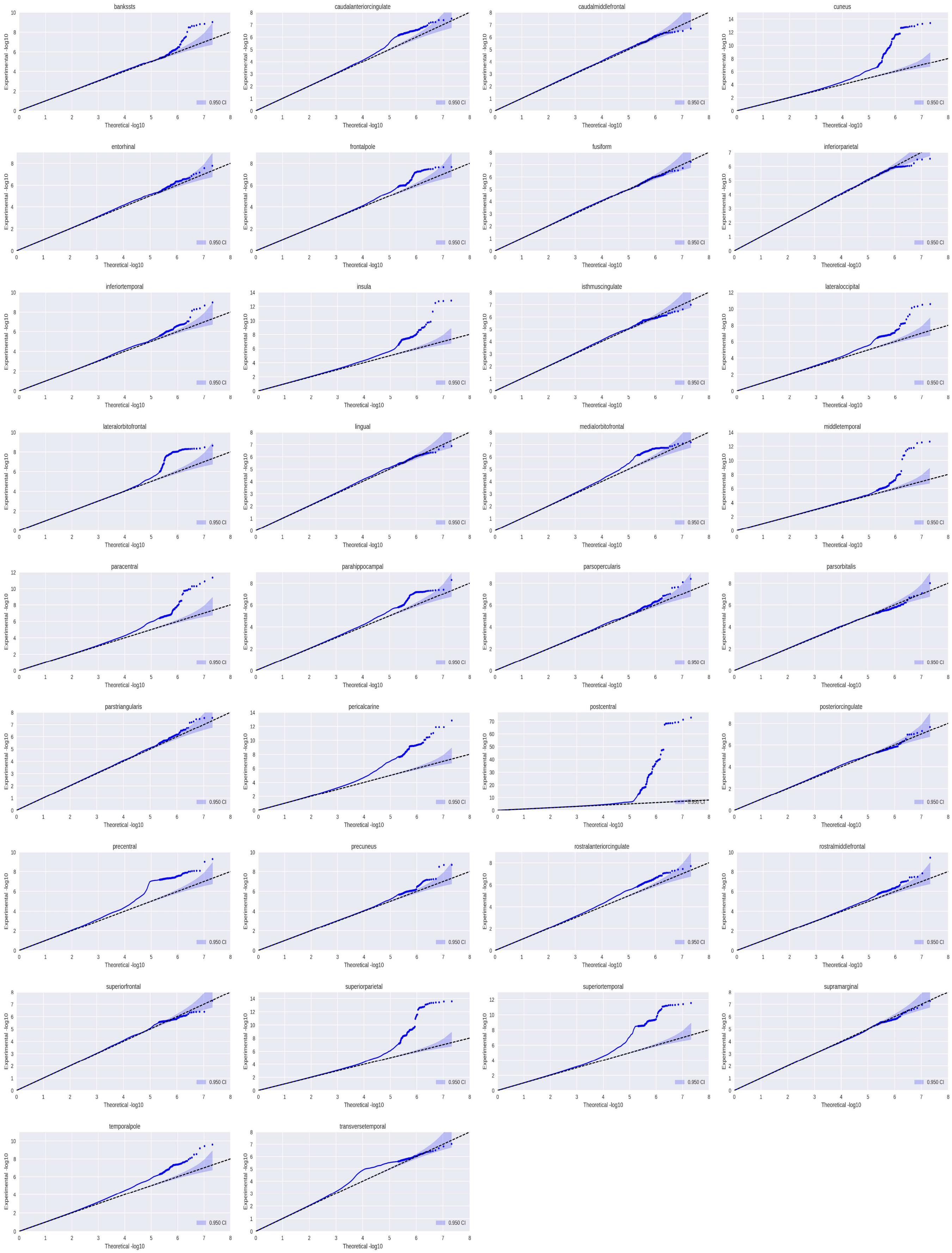

Supplementary Figure 6. QQ plots of regional cortical surface area meta-analyses.

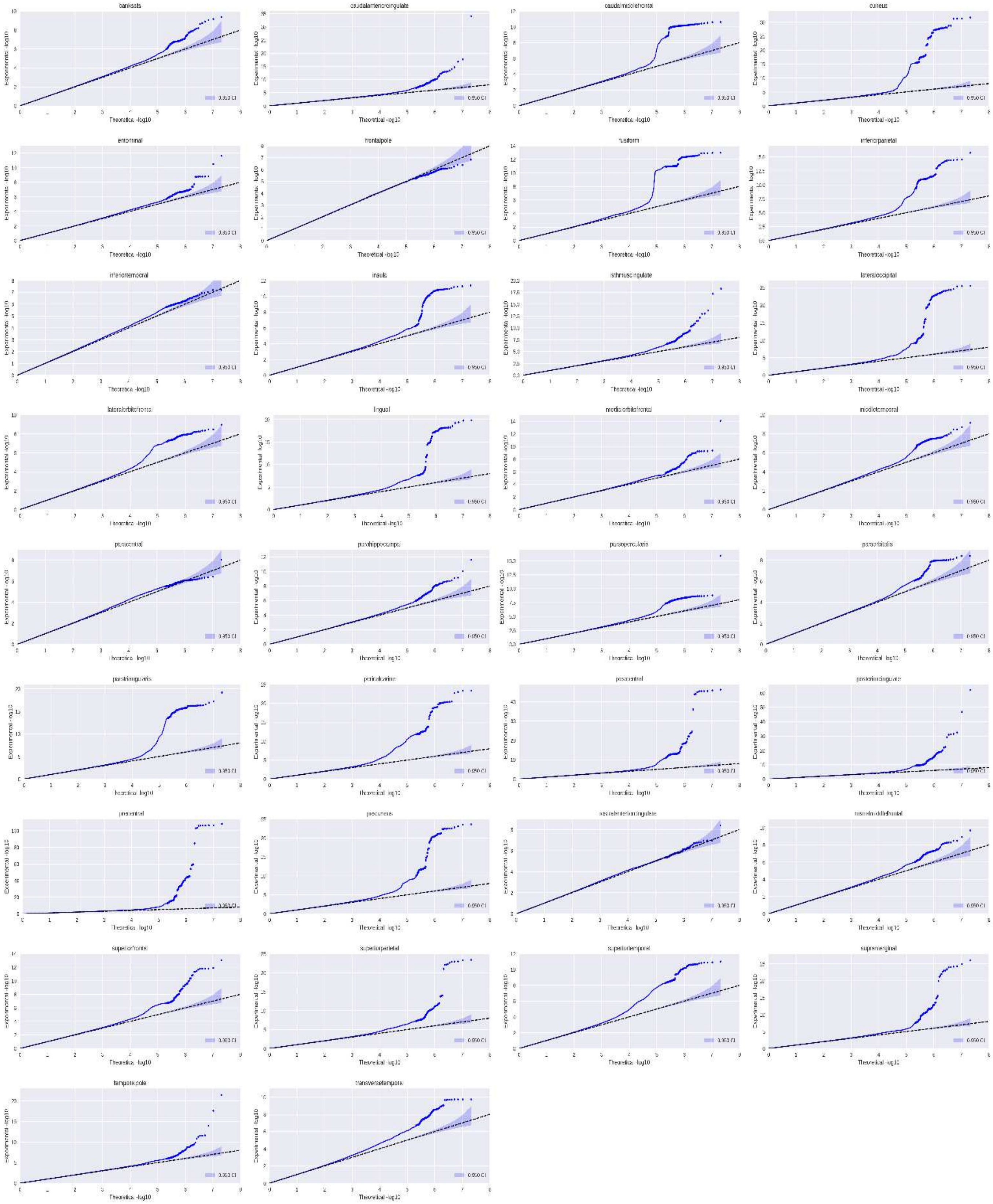

Supplementary Figure 7. QQ plots of regional cortical volume meta-analyses.

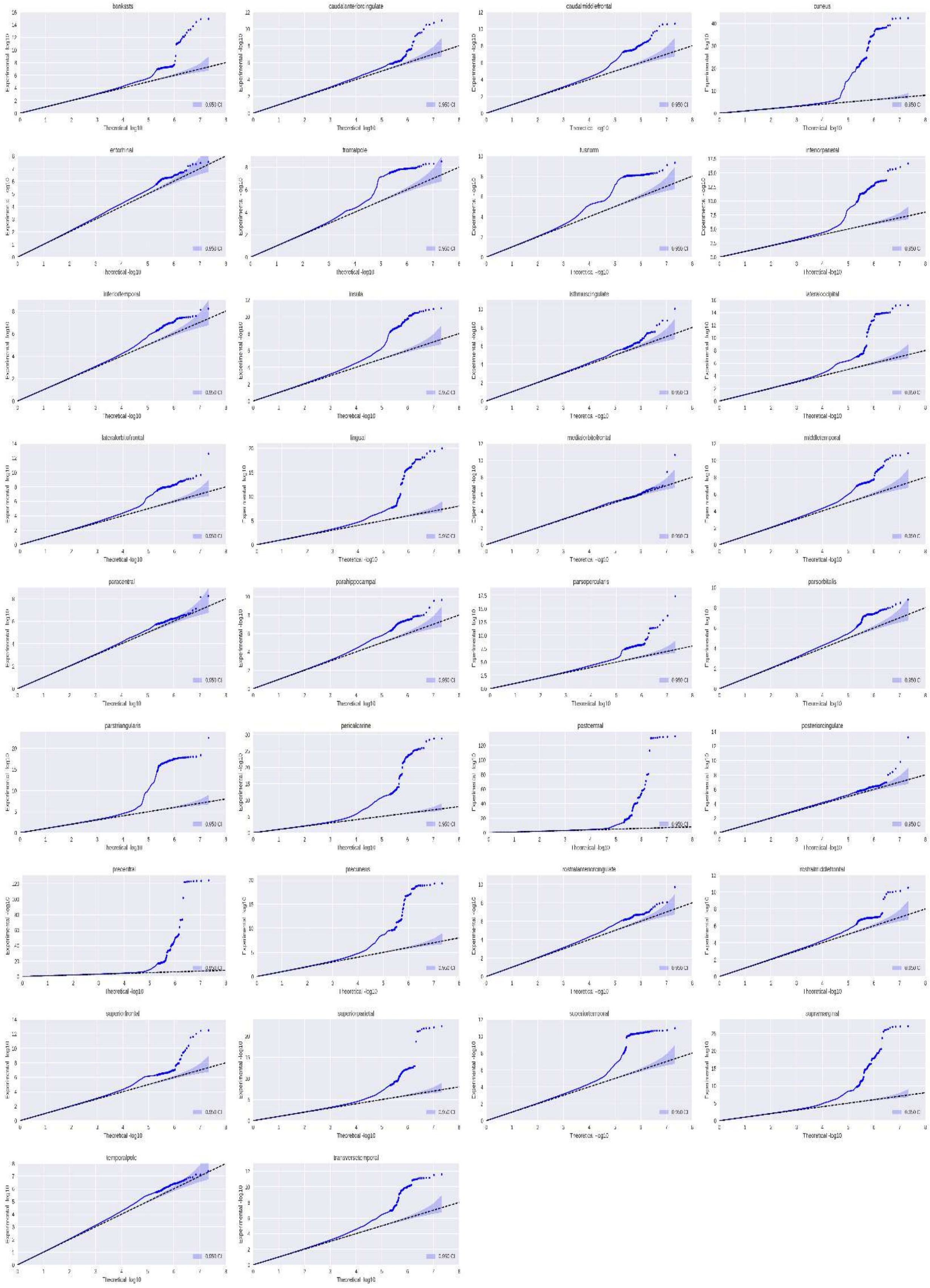

Supplementary Figure 8. Pathway analysis of 44 genes mapped to independent lead SNPs of cortical thickness.

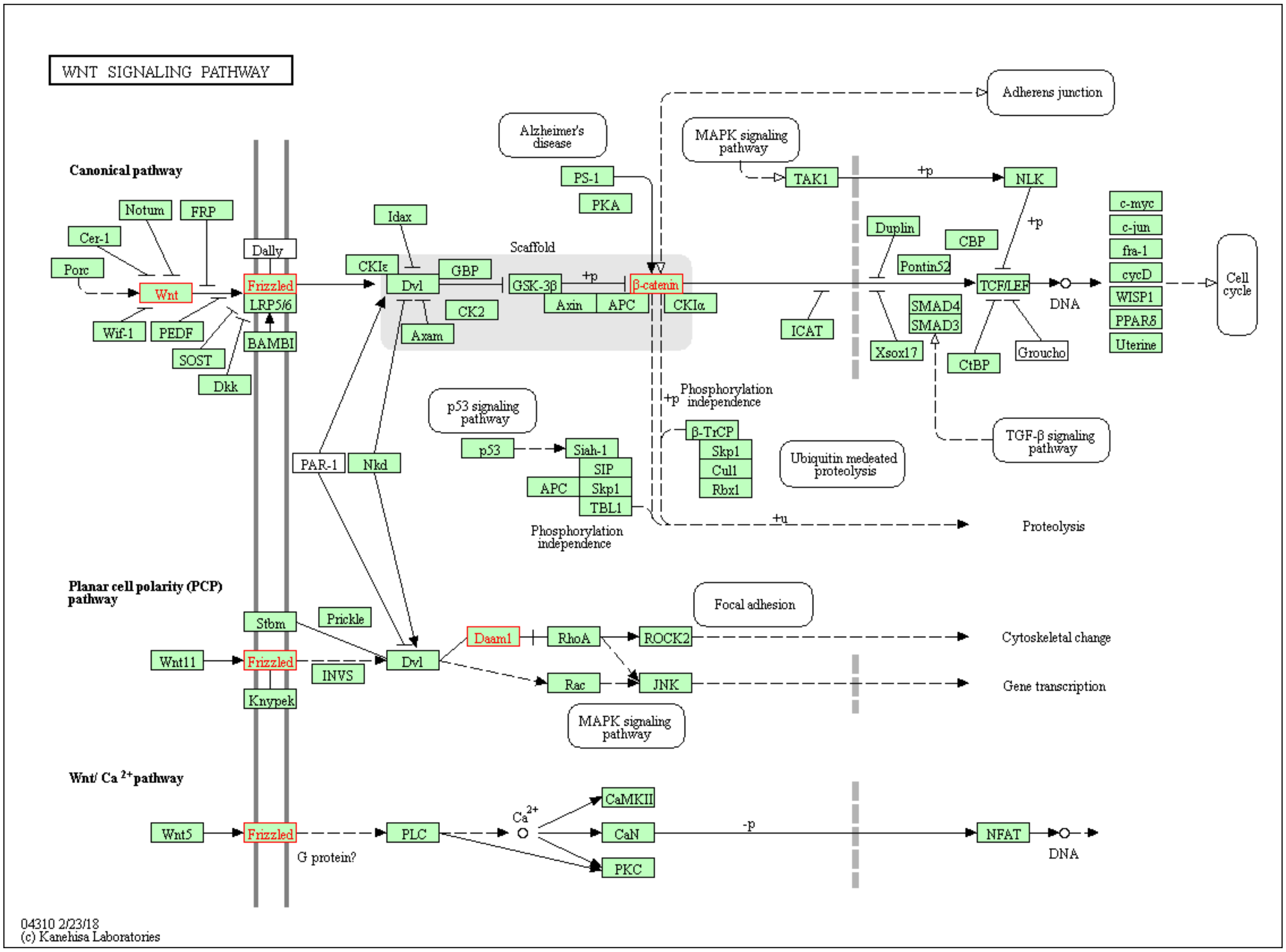

(a)

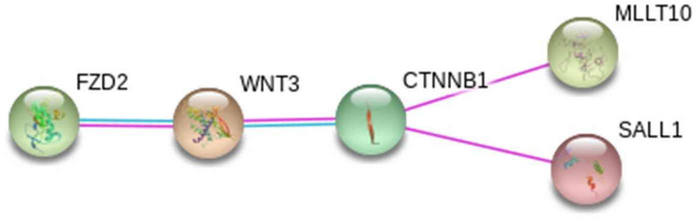

(b)

(a) The Wnt signaling pathway with 140 annotated genes based on KEGG[31] has a significant overlap with those 44 genes that were mapped to independent lead SNPs of cortical thickness (Table S17). The overlap includes *CTNNB1*, *DAAM1*, *DAAM2*, *FZD2*, and *WNT3*, which are shown in red (corrected two-sided p-value of hypergeometric test = 0.001).

(b) The protein-protein interactions have been experimentally confirmed by other scholars for three gene products in the overlap. CTNNB1 also interacts with MLLT10 and SALL1, which are among the list of 44 genes with a SNP.

Supplementary Figure 9. Pathway analysis of 105 genes mapped to independent lead SNPs of cortical surface area.

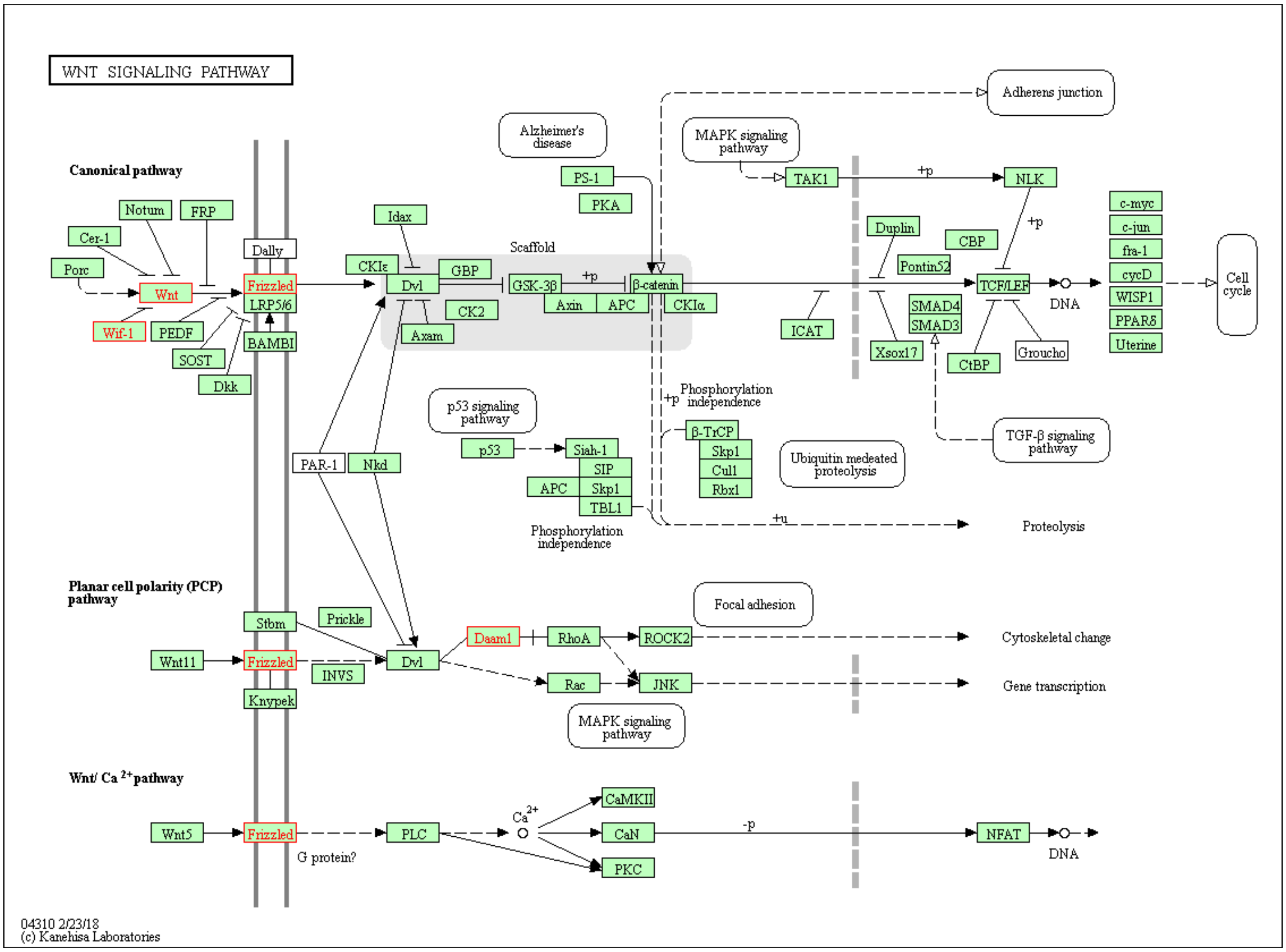

(a)

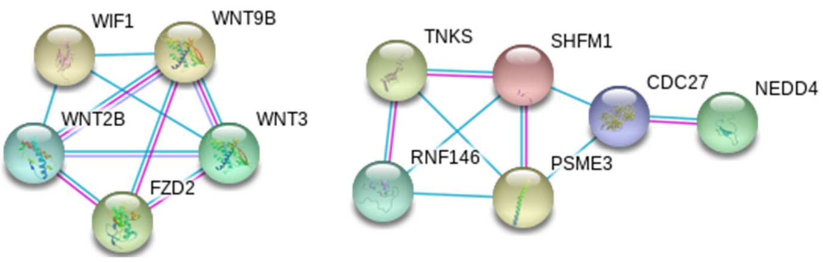

(b)

(a) The Wnt signaling pathway has a significant overlap with those 105 genes that mapped to independent lead SNPs of cortical surface area (Table S18). The overlap includes *DAAM2*, *FZD2*, *WIF1, WNT2B, WNT3,* and *WNT9B*, which are shown in red (corrected two-sided p-value of hypergeometric test = 0.005).

(b) The protein-protein interactions relate a subset of these genes in two subnetworks.

Supplementary Figure 10. Pathway analysis of 82 genes mapped to independent lead SNPs of cortical volume.

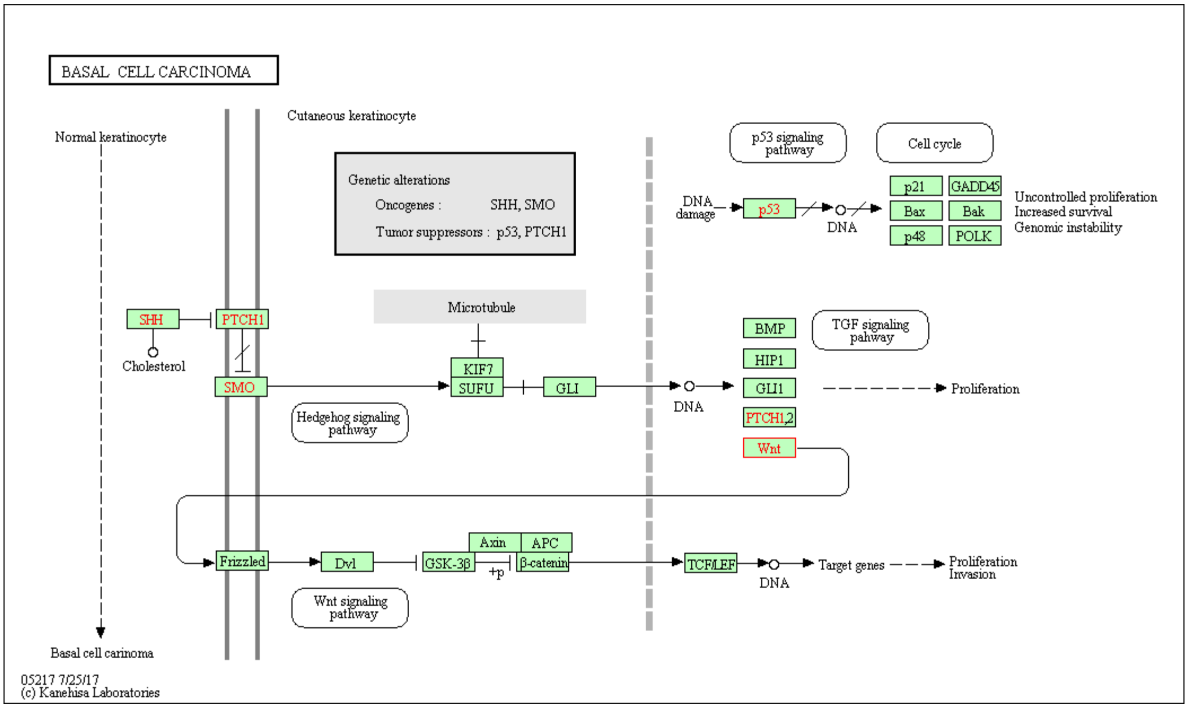

(a)

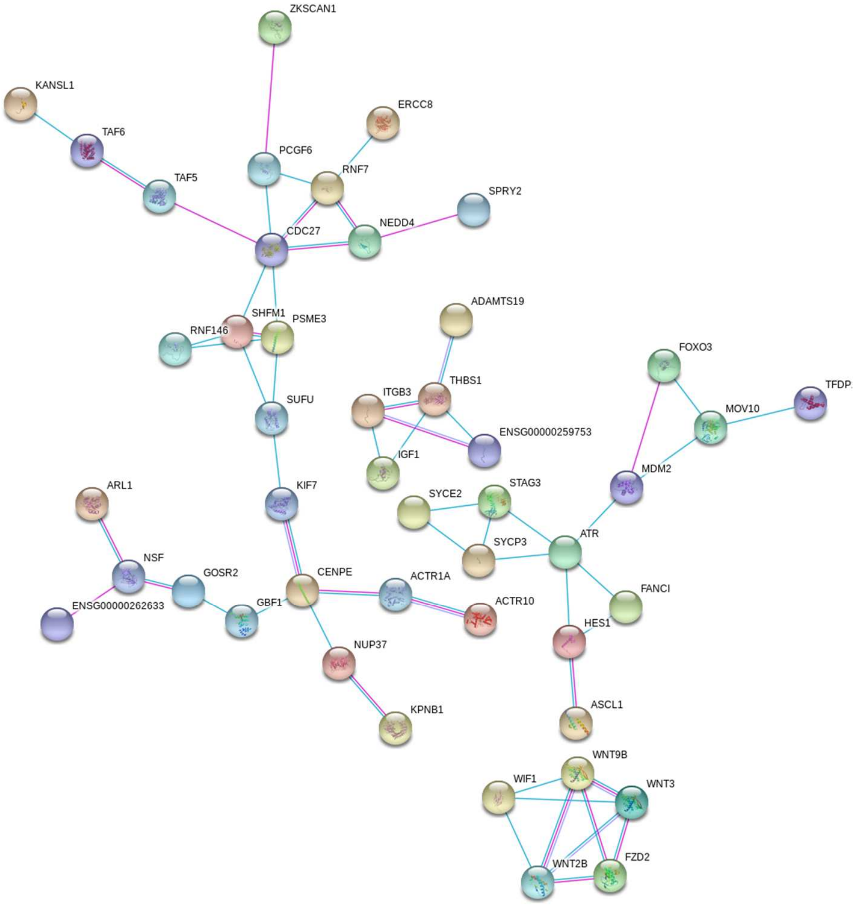

(b)

(a) The 82 genes that mapped to independent lead SNPs of cortical volume (Table S19) have overlaps with the basal cell carcinoma pathway, which has 55 annotated genes based on KEGG. The overlap includes *FZD2, SUFU, WNT2B, WNT3,* and *WNT9B* (corrected two-sided p-value of hypergeometric test = 0.04).

(b) Many of these genes have confirmed protein-protein interactions with each other.

Supplementary Figure 11. Regional heritability estimates based on common SNPs.

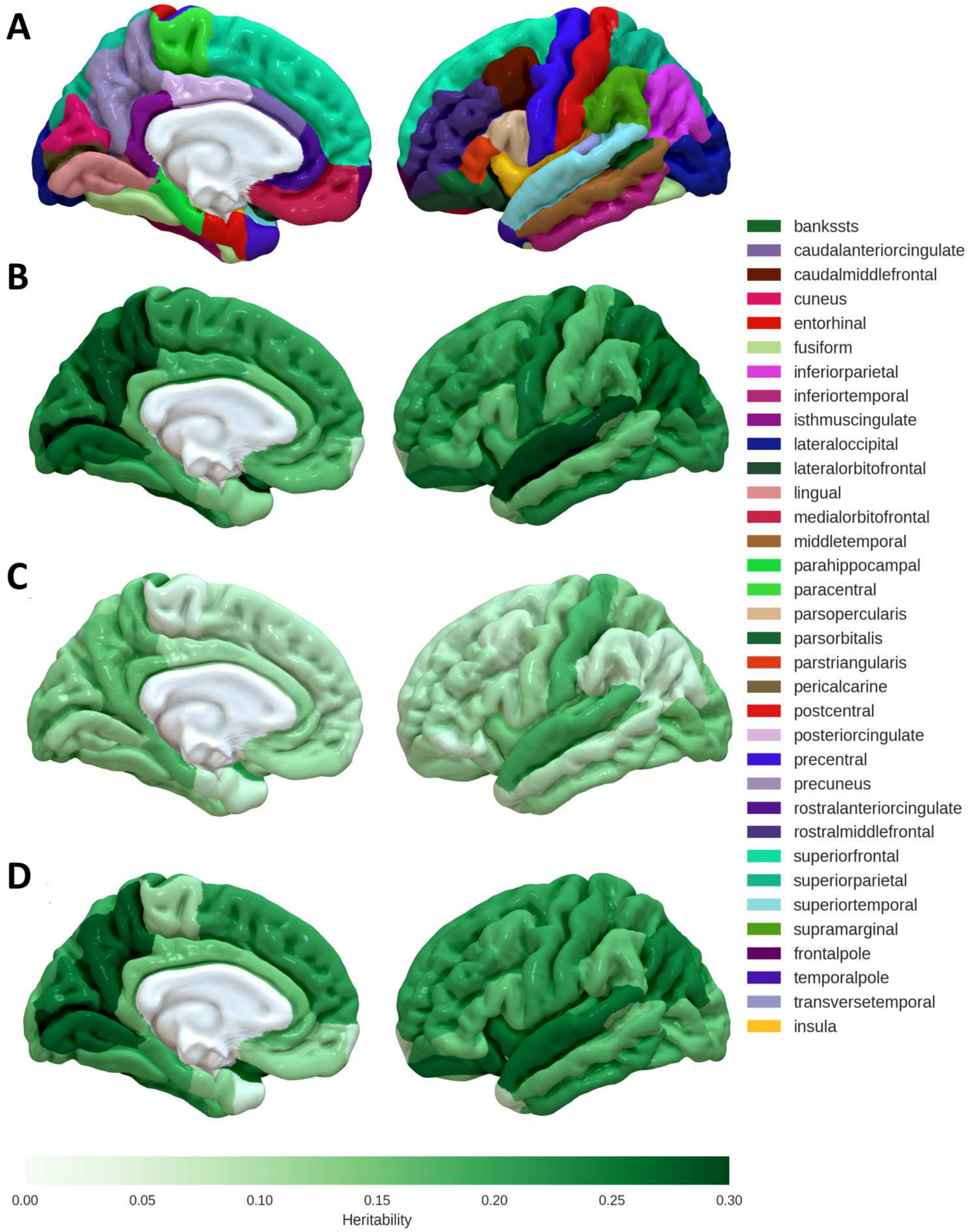

Panel (A) shows the Freesurfer segmentation. Regional heritability estimates based on common variants, calculated using LD score regression, of cortical surface area (B), cortical thickness (C) and cortical volume (D).

Supplementary Figure 12. Genetic correlation between cortical thickness, surface area and volume within cortical regions.

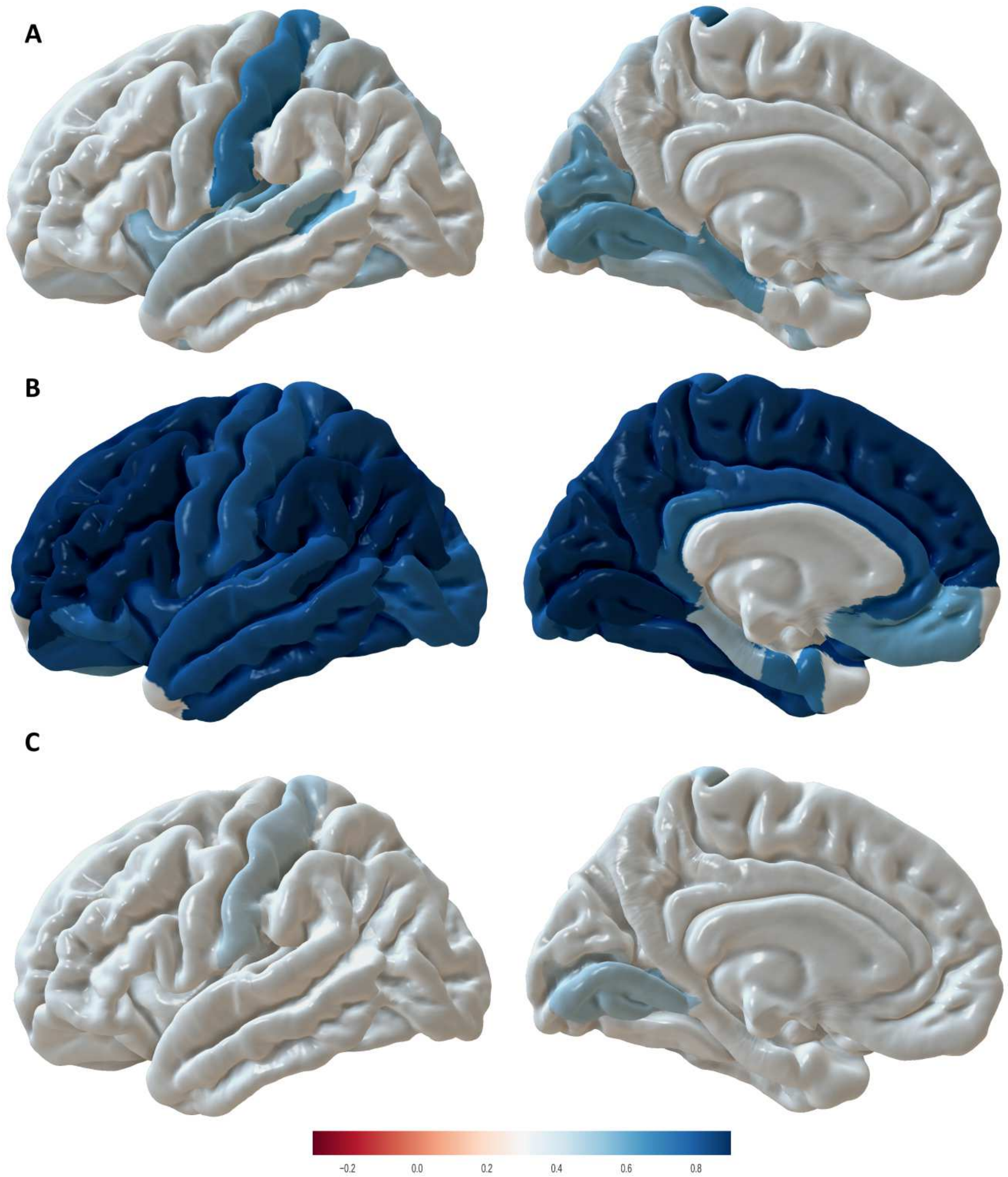

Genetic correlation between cortical thickness and volume (A), cortical volume and surface area (B), and cortical thickness and surface area (C) within cortical regions.

Supplementary Figure 13. Genetic correlation between regional cortical thickness.

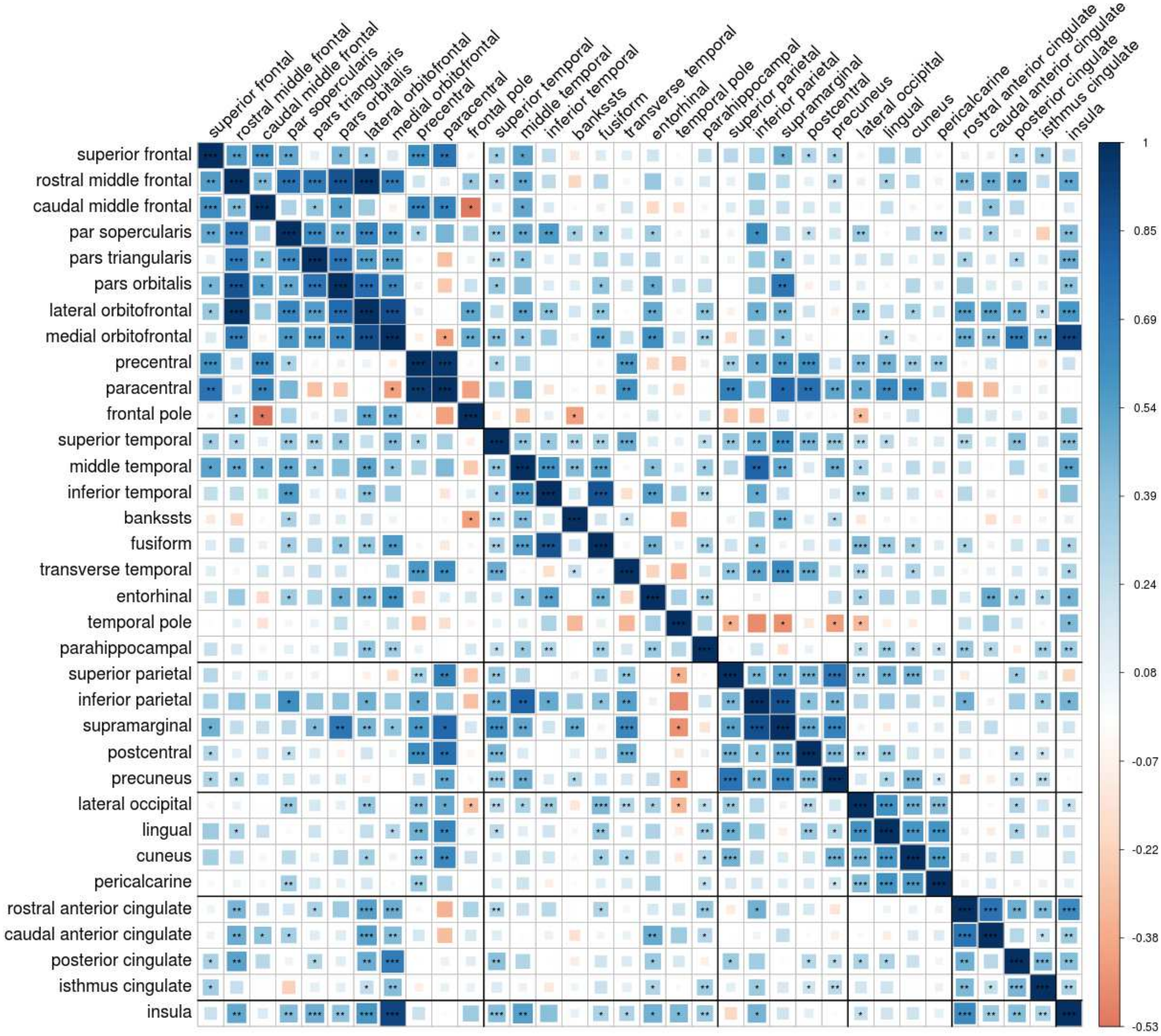

* p-value<0.05, ** p-value<0.01, *** p-value<8.91e-5 (Bonferroni correction: 0.05 (nominal significance) / 34 * (34-1) (number of regions) / 2 (half of the matrix); two-sided p-values are obtained from LD score regression; banksts=banks of the superior temporal sulcus.

Supplementary Figure 14. Genetic correlation between regional cortical surface area.

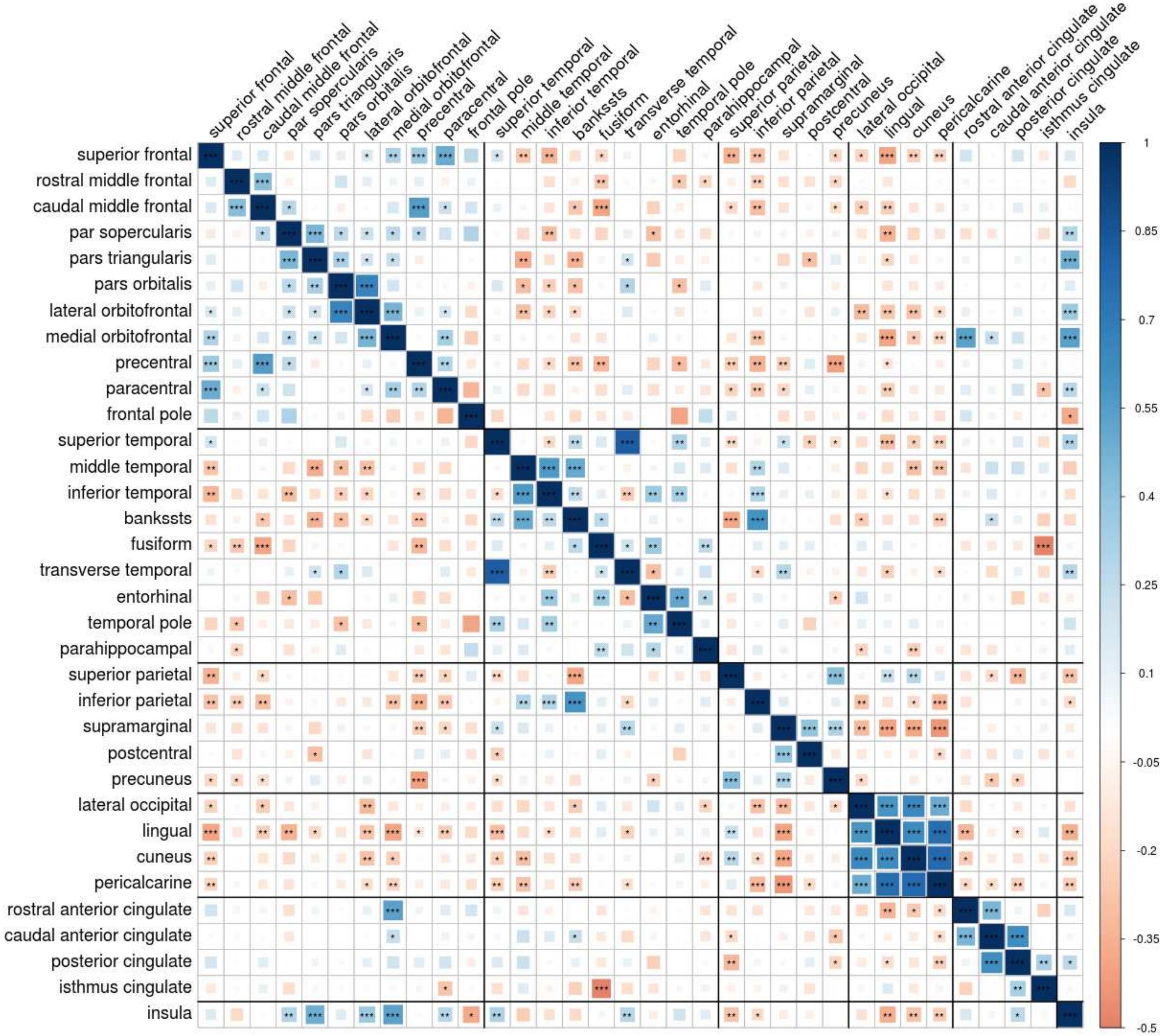

* p-value<0.05, ** p-value<0.01, *** p-value<8.91e-5 (Bonferroni correction: 0.05 (nominal significance) / 34 * (34-1) (number of regions) / 2 (half of the matrix); two-sided p-values are obtained from LD score regression; banksts=banks of the superior temporal sulcus.

Supplementary Figure 15. Genetic correlation between regional cortical volume.

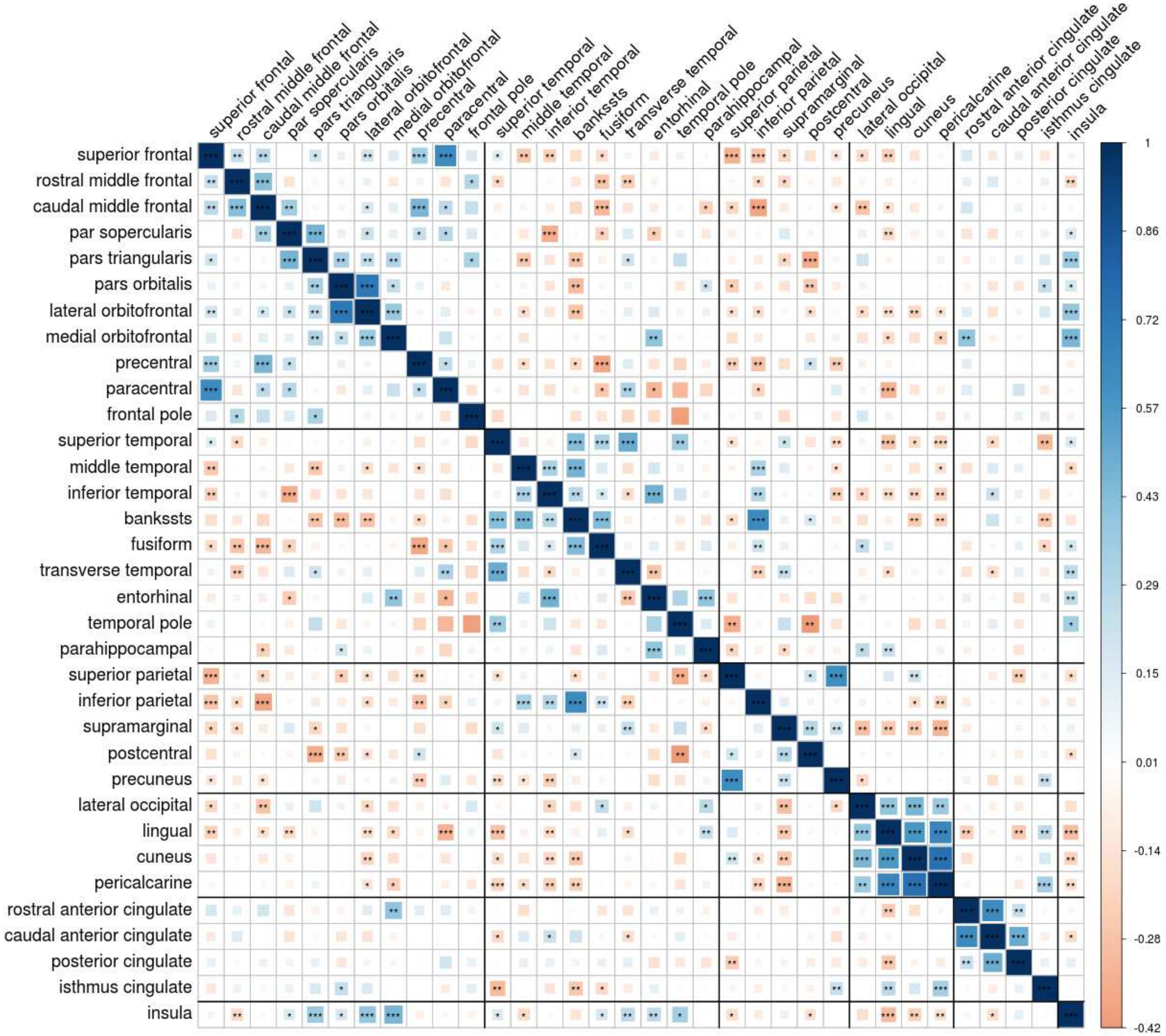

*p-value<0.05, **p-value<0.01, ***p-value<8.91e-5 (Bonferroni correction: 0.05 (nominal significance) / 34 * (34-1) (number of regions) / 2 (half of the matrix); two-sided p-values are obtained from LD score regression; banksts=banks of the superior temporal sulcus.

Supplementary Figure 16. Genetic correlation between cortical thickness and other GWAS phenotypes.

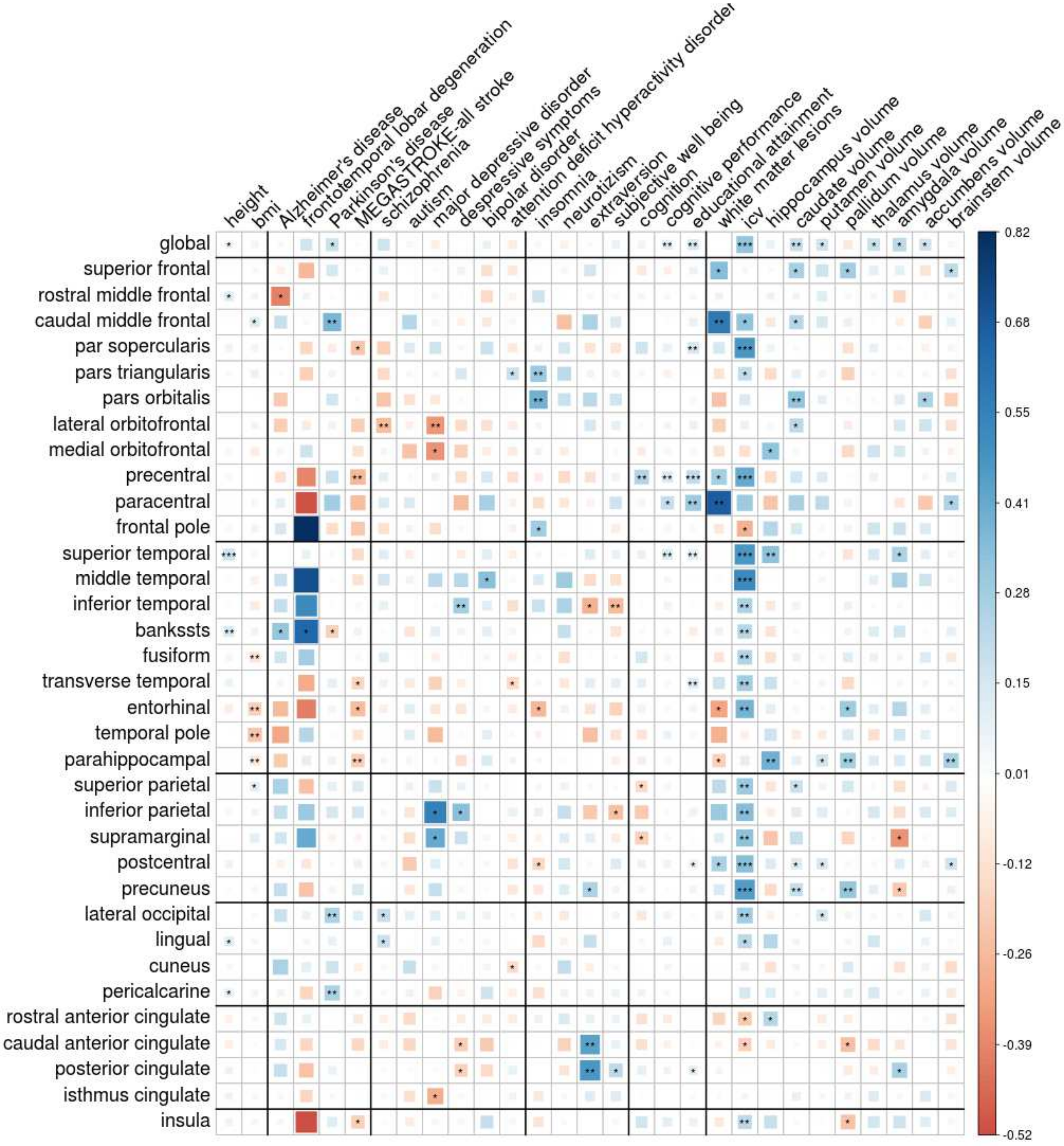

*p-value<0.05, **p-value<0.01, ***p-value=3.1*10^{-5} (Bonferroni correction: 0.05 (nominal significance) / 46 (number of independent tests)/35(number of tested traits); two-sided p-values are obtained from LD score regression; banksts=banks of the superior temporal sulcus.

Supplementary Figure 17. Genetic correlation between cortical surface area and other GWAS phenotypes.

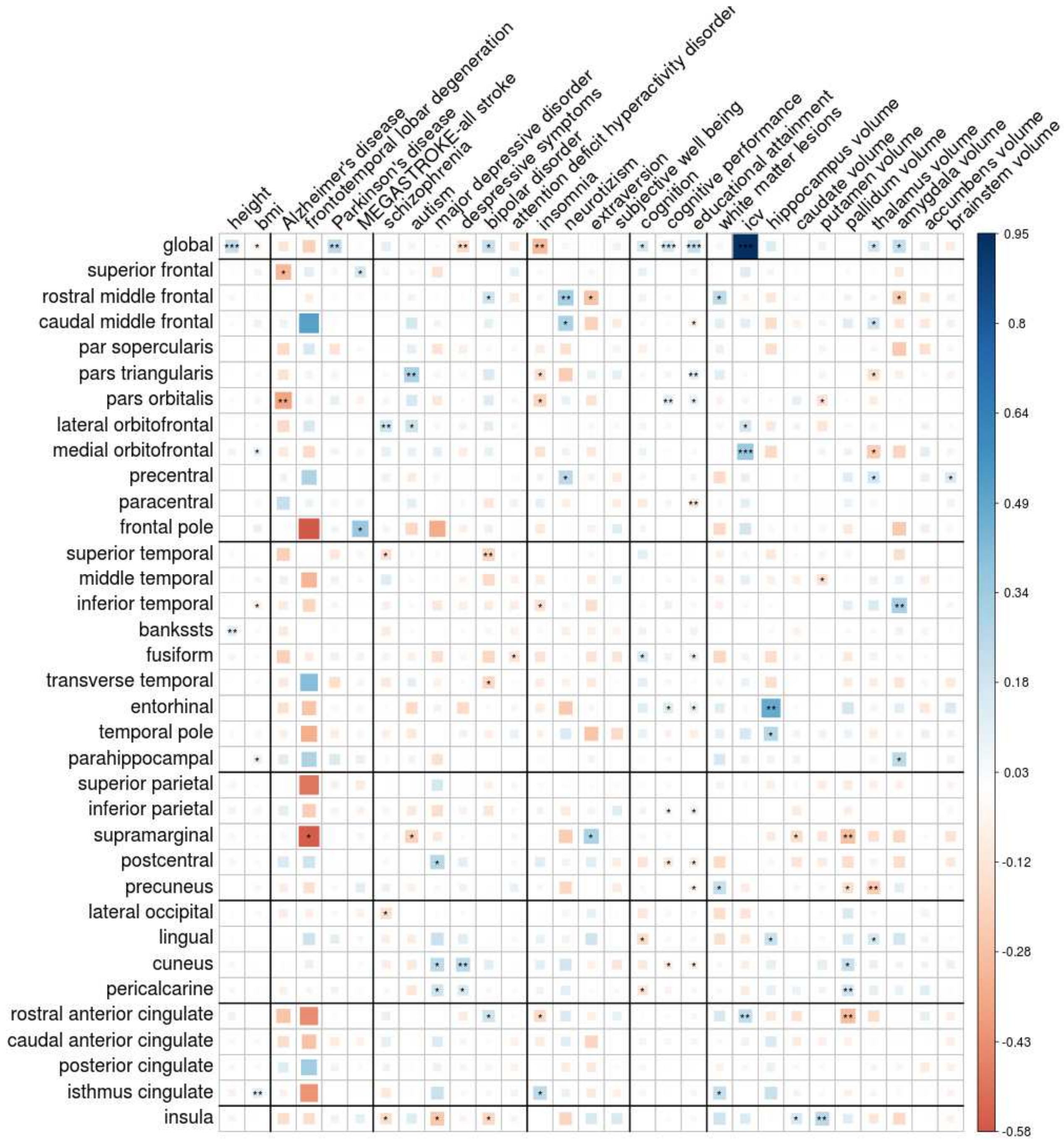

* p-value<0.05, ** p-value<0.01, p-value=3.1*10^{-5} (Bonferroni correction: 0.05 (nominal significance) / 46 (number of independent tests)/35(number of tested traits); two-sided p-values are obtained from LD score regression; banksts=banks of the superior temporal sulcus.

Supplementary Figure 18. Genetic correlation between cortical volume and other GWAS phenotypes.

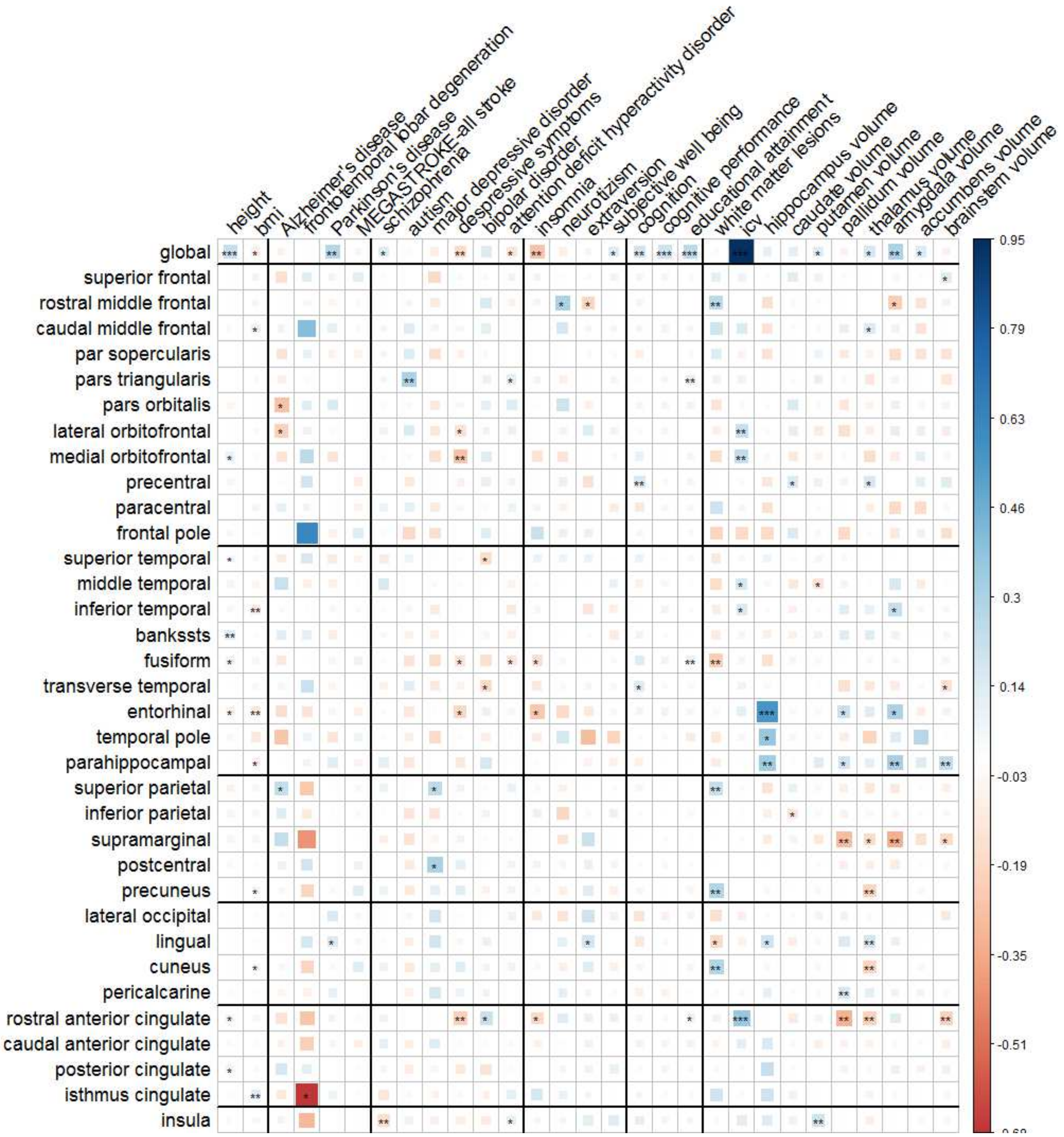

* p-value<0.05, ** p-value<0.01, *** p-value=$3.1*10^{-5}$ (Bonferroni correction: 0.05 (nominal significance) / 46 (number of independent tests)/35(number of tested traits); two-sided p-values are obtained from LD score regression; banksts=banks of the superior temporal sulcus.

Supplementary Notes

Supplementary Note 1: Acknowledgements

Cohorts for Heart and Aging Research in Genomic Epidemiology (CHARGE)

Infrastructure for the CHARGE Consortium is supported in part by the National Heart, Lung, and Blood Institute grant HL105756 and for the neuroCHARGE phenotype working group through the National Institute on Aging grant AG033193.

Atherosclerosis Risk in Communities Study (ARIC)

The Atherosclerosis Risk in Communities (ARIC) study is carried out as a collaborative study supported by the National Heart, Lung, and Blood Institute (NHLBI) contracts (HHSN268201100005C, HHSN268201100006C, HHSN268201100007C, HHSN268201100008C, HHSN268201100009C, HHSN268201100010C, HHSN268201100011C, and HHSN268201100012C). The authors thank the staff and participants of the ARIC study for their important contributions. Funding support for "Building on GWAS for NHLBI-diseases: the U.S. CHARGE consortium" was provided by the NIH through the American Recovery and Reinvestment Act of 2009 (ARRA) (5RC2HL102419). This project was also funded from R01-NS087541.

Austrian Stroke Prevention Family (ASPS) / Austrian Stroke Prevention Family Study (ASPS-Fam)

The authors thank the staff and the participants for their valuable contributions. We thank Birgit Reinhart for her long-term administrative commitment, Elfi Hofer for the technical assistance at creating the DNA bank, Ing. Johann Semmler and Anita Harb for DNA sequencing and DNA analyses by TaqMan assays and Irmgard Poelzl for supervising the quality management processes after ISO9001 at the biobanking and DNA analyses.
The Medical University of Graz and the Steiermärkische Krankenanstaltengesellschaft support the databank of the ASPS/ASPS-Fam. The research reported in this article was funded by the Austrian Science Fund (FWF) grant numbers PI904, P20545-P05 and P13180 and supported by the Austrian National Bank Anniversary Fund, P15435 and the Austrian Ministry of Science under the aegis of the EU Joint Programme-Neurodegenerative Disease Research (JPND)-www.jpnd.eu.

Cardiovascular Health Study (CHS)

Cardiovascular Health Study: This CHS research was supported by NHLBI contracts HHSN268201200036C, HHSN268200800007C, HHSN268201800001C, N01HC55222, N01HC85079, N01HC85080, N01HC85081, N01HC85082, N01HC85083, N01HC85086, N01HC15103, HHSN268200960009C; and NHLBI grants U01HL080295, R01HL087652, R01HL105756, R01HL103612, R01HL120393, and U01HL130114 with additional

contribution from the National Institute of Neurological Disorders and Stroke (NINDS). Additional support was provided through R01AG023629, and R01AG033193 from the National Institute on Aging (NIA). A full list of principal CHS investigators and institutions can be found at CHS-NHLBI.org. The provision of genotyping data was supported in part by the National Center for Advancing Translational Sciences, CTSI grant UL1TR001881, and the National Institute of Diabetes and Digestive and Kidney Disease Diabetes Research Center (DRC) grant DK063491 to the Southern California Diabetes Endocrinology Research Center. The content is solely the responsibility of the authors and does not necessarily represent the official views of the National Institutes of Health.

Erasmus Rucphen Family Study (ERF)

Erasmus Rucphen Family (ERF) was supported by the Consortium for Systems Biology (NCSB), both within the framework of the Netherlands Genomics Initiative (NGI)/Netherlands Organisation for Scientific Research (NWO). ERF study as a part of EUROSPAN (European Special Populations Research Network) was supported by European Commission FP6 STRP grant number 018947 (LSHG-CT-2006-01947) and also received funding from the European Community's Seventh Framework Programme (FP7/2007-2013)/grant agreement HEALTH-F4-2007-201413 by the European Commission under the programme "Quality of Life and Management of the Living Resources" of 5th Framework Programme (No. QLG2-CT-2002-01254) as well as FP7 project EUROHEADPAIN (nr 602633). High-throughput analysis of the ERF data was supported by joint grant from Netherlands Organisation for Scientific Research and the Russian Foundation for Basic Research (NWO-RFBR 047.017.043). High throughput metabolomics measurements of the ERF study has been supported by BBMRI-NL (Biobanking and Biomolecular Resources Research Infrastructure Netherlands).

Framingham Heart Study (FHS)

This work was supported by the National Heart, Lung and Blood Institute's Framingham Heart Study (Contract No. N01-HC-25195 and No. HHSN268201500001I) and its contract with Affymetrix, Inc. for genotyping services (Contract No. N02-HL-6-4278). A portion of this research utilized the Linux Cluster for Genetic Analysis (LinGA-II) funded by the Robert Dawson Evans Endowment of the Department of Medicine at Boston University School of Medicine and Boston Medical Center. This study was also supported by grants from the National Institute of Aging (R01s AG033040, AG033193, AG054076, AG049607, AG008122, AG016495; and U01-AG049505) and the National Institute of Neurological Disorders and Stroke (R01-NS017950). We would like to thank the dedication of the Framingham Study participants, as well as the Framingham Study team, especially investigators and staff from the Neurology group, for their contributions to data collection. Dr. DeCarli is supported by the Alzheimer's Disease Center (P30 AG 010129). The views expressed in this manuscript are those of the authors and do not necessarily represent the views of the National Heart, Lung, and Blood Institute; the National Institutes of Health; or the U.S. Department of Health and Human Services.

Lothian Birth Cohort 1936 (LBC1936)

This project is funded by the Age UK's Disconnected Mind programme (http://www.disconnectedmind.ed.ac.uk) and also by Research Into Ageing (Refs. 251 and 285). The whole genome association part of the study was funded by the Biotechnology and Biological Sciences Research Council (BBSRC; Ref. BB/F019394/1). Analysis of the brain images was funded by the Medical Research Council Grants G1001401 and 8200 and MR/M01311/1. The imaging was performed at the Brain Research Imaging Centre, The University of Edinburgh (http://www.bric.ed.ac.uk), a centre in the SINAPSE Collaboration (http://www.sinapse.ac.uk). The work was undertaken by The University of Edinburgh Centre for Cognitive Ageing and Cognitive Epidemiology (http://www.ccace.ed.ac.uk), part of the cross council Lifelong Health and Wellbeing Initiative (Ref. G0700704/84698). Funding from the BBSRC, Medical Research Council (MR/K026992/1) and Scottish Funding Council through the SINAPSE Collaboration is gratefully acknowledged.

We thank the LBC1936 participants and research team members. We also thank the nurses and staff at the Wellcome Trust Clinical Research Facility (http://www.wtcrf.ed.ac.uk), where subjects were tested and the genotyping was performed.

LIFE-Adult

LIFE-Adult is funded by the Leipzig Research Center for Civilization Diseases (LIFE). LIFE is an organizational unit affiliated to the Medical Faculty of the University of Leipzig. LIFE is funded by means of the European Union, by the European Regional Development Fund (ERDF) and by funds of the Free State of Saxony within the framework of the excellence initiative. This work was also funded by the Deutsche Forschungsgemeinschaft (Grant Number: CRC 1052 "Obesity mechanisms" project A1 to AV) and by the Max Planck Society.

Sydney Memory and Ageing Study (MAS)

MAS is funded by the Australian National Health and Medical Research Council (NHMRC)/Australian Research Council Strategic Award (Grant 401162), NHMRC Project grant 1405325.

We would like to gratefully acknowledge and thank the Sydney MAS participants and supporters and the Sydney MAS Research Team.

Older Australian Twin Study (OATS)

OATS is funded by the Australian National Health and Medical Research Council (NHMRC)/Australian Research Council Strategic Award (Grant 401162), NHMRC Program Grants (350833, 568969, 109308)

We would like thank and gratefully acknowledge the OATS participants, their supporters and the OATS Research Team.

Rotterdam Study (RSI, RSII, RSIII)

The Rotterdam Study is funded by Erasmus Medical Center and Erasmus University, Rotterdam, Netherlands Organization for the Health Research and Development (ZonMw), the Research Institute for Diseases in the Elderly (RIDE), the Ministry of Education, Culture and Science, the Ministry for Health, Welfare and Sports, the European Commission (DG XII), and the Municipality of Rotterdam. The authors are grateful to the study participants, the staff from the Rotterdam Study and the participating general practitioners and pharmacists. The generation and management of GWAS genotype data for the Rotterdam Study (RS I, RS II, RS III) were executed by the Human Genotyping Facility of the Genetic Laboratory of the Department of Internal Medicine, Erasmus MC, Rotterdam, The Netherlands. The GWAS datasets are supported by the Netherlands Organisation of Scientific Research NWO Investments (nr. 175.010.2005.011, 911-03-012), the Genetic Laboratory of the Department of Internal Medicine, Erasmus MC, the Research Institute for Diseases in the Elderly (014-93-015; RIDE2), the Netherlands Genomics Initiative (NGI)/Netherlands Organisation for Scientific Research (NWO) Netherlands Consortium for Healthy Aging (NCHA), project nr. 050-060-810. We thank Pascal Arp, Mila Jhamai, Marijn Verkerk, Lizbeth Herrera and Marjolein Peters, and Carolina Medina-Gomez, for their help in creating the GWAS database, and Karol Estrada, Yurii Aulchenko, and Carolina Medina-Gomez, for the creation and analysis of imputed data. This work has been performed as part of the CoSTREAM project (www.costream.eu) and ORACLE project, and has received funding from the European Union's Horizon 2020 research and innovation programme under grant agreement No 667375 and No 678543. HHHA was supported by ZonMW grant number 916.19.151.

Study of Health in Pomerania (SHIP) / Study of Health in Pomerania Trend (SHIP-Trend)

SHIP is part of the Community Medicine Research net of the University of Greifswald, Germany, which is funded by the Federal Ministry of Education and Research (grants no. 01ZZ9603, 01ZZ0103, and 01ZZ0403), the Ministry of Cultural Affairs as well as the Social Ministry of the Federal State of Mecklenburg-West Pomerania, and the network 'Greifswald Approach to Individualized Medicine (GANI_MED)' funded by the Federal Ministry of Education and Research (grant 03IS2061A). Genome-wide data have been supported by the Federal Ministry of Education and Research (grant no. 03ZIK012) and a joint grant from Siemens Healthineers, Erlangen, Germany and the Federal State of Mecklenburg- West Pomerania. Whole-body MR imaging was supported by a joint grant from Siemens Healthineers, Erlangen, Germany and the Federal State of Mecklenburg West Pomerania. The University of Greifswald is a member of the Caché Campus program of the InterSystems GmbH.

Saguenay Youth Study (SYS)

The Saguenay Youth Study has been funded by the Canadian Institutes of Health Research (TP, ZP), Heart and Stroke Foundation of Canada (ZP), and the Canadian Foundation for Innovation (ZP). We thank all families who

took part in the Saguenay Youth Study. SYS is supported by the Canadian Institutes of Health Research: NET54015, NRF86678, TMH109788.

Three-City Dijon (3C-Dijon)

The Three City (3C) Study is conducted under a partnership agreement among the Institut National de la Santé et de la Recherche Médicale (INSERM), the University of Bordeaux, and Sanofi-Aventis. The Fondation pour la Recherche Médicale funded the preparation and initiation of the study. The 3C Study is also supported by the Caisse Nationale Maladie des Travailleurs Salariés, Direction Générale de la Santé, Mutuelle Générale de l'Education Nationale (MGEN), Institut de la Longévité, Conseils Régionaux of Aquitaine and Bourgogne, Fondation de France, and Ministry of Research–INSERM Programme "Cohortes et collections de données biologiques." Christophe Tzourio and Stéphanie Debette have received investigator-initiated research funding from the French National Research Agency (ANR) and from the Fondation Leducq. Stéphanie Debette is supported by a starting grant from the European Research Council (SEGWAY) and a grant from the Joint Programme of Neurodegenerative Disease research (BRIDGET), from the European Union's Horizon 2020 research and innovation programme under grant agreements No 643417 & No 640643, and by the Initiative of Excellence of Bordeaux University. We thank Dr. Anne Boland (CNG) for her technical help in preparing the DNA samples for analyses. This work was supported by the National Foundation for Alzheimer's disease and related disorders, the Institut Pasteur de Lille, the labex DISTALZ and the Centre National de Génotypage.

United Kingdom Biobank (UKBB)

This research has been conducted using the UK Biobank Resource under Application Number "23509".

Vietnam Era Twin Study of Aging (VETSA)

This work was supported by US National Institutes of Health grants AG018386, AG022381, AG022982, AG050595, AG018384, AG046413, AG047903, DA025109, DA023549, DA18673, HD050735, and U 54EB020403, and the VA San Diego Center of Excellence for Stress and Mental Health Healthcare System. The content is the responsibility of the authors and does not necessarily represent official views of the NIA, NIH, or VA. The Cooperative Studies Program of the U.S. Department of Veterans Affairs provided financial support for development and maintenance of the Vietnam Era Twin Registry. We would also like to acknowledge the continued cooperation and participation of the members of the VET Registry and their families.

ENIGMA

The study was supported in part by grant U54 EB020403 from the NIH Big Data to Knowledge (BD2K) Initiative, a cross-NIH partnership. Additional support was provided by R01MH116147, P41 EB015922, RF1AG051710, RF1 AG041915 (to P.T.), by P01 AG026572, R01 AG059874 and by R01 MH117601 (to N.J. and L.S.). S.E.M. was

funded by an NHMRC Senior Research Fellowship (APP1103623). L.C.-C. was supported by a QIMR Berghofer Fellowship.

Supplementary References

1 The Atherosclerosis Risk in Communities (ARIC) Study: design and objectives. The ARIC investigators. *American journal of epidemiology* **129**, 687-702 (1989).

2 Schmidt, R. *et al.* Assessment of cerebrovascular risk profiles in healthy persons: definition of research goals and the Austrian Stroke Prevention Study (ASPS). *Neuroepidemiology* **13**, 308-313, doi:10.1159/000110396 (1994).

3 Schmidt, R., Fazekas, F., Kapeller, P., Schmidt, H. & Hartung, H. P. MRI white matter hyperintensities: three-year follow-up of the Austrian Stroke Prevention Study. *Neurology* **53**, 132-139 (1999).

4 Seiler, S. *et al.* Magnetization transfer ratio relates to cognitive impairment in normal elderly. *Frontiers in aging neuroscience* **6**, 263, doi:10.3389/fnagi.2014.00263 (2014).

5 Ghadery, C. *et al.* R2* mapping for brain iron: associations with cognition in normal aging. *Neurobiology of aging* **36**, 925-932, doi:10.1016/j.neurobiolaging.2014.09.013 (2015).

6 Fried, L. P. *et al.* The Cardiovascular Health Study: design and rationale. *Annals of epidemiology* **1**, 263-276 (1991).

7 Isaacs, A. *et al.* Heritabilities, apolipoprotein E, and effects of inbreeding on plasma lipids in a genetically isolated population: the Erasmus Rucphen Family Study. *European journal of epidemiology* **22**, 99-105, doi:10.1007/s10654-006-9103-0 (2007).

8 Sayed-Tabatabaei, F. A. *et al.* Heritability of the function and structure of the arterial wall: findings of the Erasmus Rucphen Family (ERF) study. *Stroke* **36**, 2351-2356, doi:10.1161/01.STR.0000185719.66735.dd (2005).

9 Dawber, T. R. & Kannel, W. B. The Framingham study. An epidemiological approach to coronary heart disease. *Circulation* **34**, 553-555 (1966).

10 Feinleib, M., Kannel, W. B., Garrison, R. J., McNamara, P. M. & Castelli, W. P. The Framingham Offspring Study. Design and preliminary data. *Preventive medicine* **4**, 518-525 (1975).

11 Splansky, G. L. *et al.* The Third Generation Cohort of the National Heart, Lung, and Blood Institute's Framingham Heart Study: design, recruitment, and initial examination. *American journal of epidemiology* **165**, 1328-1335, doi:10.1093/aje/kwm021 (2007).

12 Deary, I. J. *et al.* The Lothian Birth Cohort 1936: a study to examine influences on cognitive ageing from age 11 to age 70 and beyond. *BMC geriatrics* **7**, 28, doi:10.1186/1471-2318-7-28 (2007).

13 Wardlaw, J. M. *et al.* Brain aging, cognition in youth and old age and vascular disease in the Lothian Birth Cohort 1936: rationale, design and methodology of the imaging protocol. *International journal of stroke : official journal of the International Stroke Society* **6**, 547-559, doi:10.1111/j.1747-4949.2011.00683.x (2011).

14 Houlihan, L. M. *et al.* Common variants of large effect in F12, KNG1, and HRG are associated with activated partial thromboplastin time. *American journal of human genetics* **86**, 626-631, doi:10.1016/j.ajhg.2010.02.016 (2010).

15 Loeffler, M. *et al.* The LIFE-Adult-Study: objectives and design of a population-based cohort study with 10,000 deeply phenotyped adults in Germany. *BMC public health* **15**, 691, doi:10.1186/s12889-015-1983-z (2015).

16 Sachdev, P. S. *et al.* The Sydney Memory and Ageing Study (MAS): methodology and baseline medical and neuropsychiatric characteristics of an elderly epidemiological non-demented cohort of Australians aged 70-90 years. *International psychogeriatrics* **22**, 1248-1264, doi:10.1017/S1041610210001067 (2010).

17 Sachdev, P. S. *et al.* A comprehensive neuropsychiatric study of elderly twins: the Older Australian Twins Study. *Twin research and human genetics : the official journal of the International Society for Twin Studies* **12**, 573-582, doi:10.1375/twin.12.6.573 (2009).

18 Hofman, A. *et al.* The Rotterdam Study: 2016 objectives and design update. *European journal of epidemiology* **30**, 661-708, doi:10.1007/s10654-015-0082-x (2015).

19 Volzke, H. *et al.* Cohort profile: the study of health in Pomerania. *International journal of epidemiology* **40**, 294-307, doi:10.1093/ije/dyp394 (2011).

20 Group, C. S. Vascular factors and risk of dementia: design of the Three-City Study and baseline characteristics of the study population. *Neuroepidemiology* **22**, 316-325, doi:10.1159/000072920 (2003).

21 Godin, O. *et al.* White matter lesions as a predictor of depression in the elderly: the 3C-Dijon study. *Biological psychiatry* **63**, 663-669, doi:10.1016/j.biopsych.2007.09.006 (2008).

22 Soumare, A. *et al.* White matter lesions volume and motor performances in the elderly. *Annals of neurology* **65**, 706-715, doi:10.1002/ana.21674 (2009).

23 Bycroft, C. *et al.* Genome-wide genetic data on ~500,000 UK Biobank participants. *bioRxiv*, doi:10.1101/166298 (2017).

24 Alfaro-Almagro, F. *et al.* Image Processing and Quality Control for the first 10,000 Brain Imaging Datasets from UK Biobank. *bioRxiv*, doi:10.1101/130385 (2017).

25 Miller, K. L. *et al.* Multimodal population brain imaging in the UK Biobank prospective epidemiological study. *Nat Neurosci* **19**, 1523-1536, doi:10.1038/nn.4393 http://www.nature.com/neuro/journal/v19/n11/abs/nn.4393.html#supplementary-information (2016).

26 Tsuang, M. T. *et al.* Genetic influences on DSM-III-R drug abuse and dependence: a study of 3,372 twin pairs. *American journal of medical genetics* **67**, 473-477, doi:10.1002/(SICI)1096-8628(19960920)67:5<473::AID-AJMG6>3.0.CO;2-L (1996).

27 Kremen, W. S., Franz, C. E. & Lyons, M. J. VETSA: the Vietnam Era Twin Study of Aging. *Twin research and human genetics : the official journal of the International Society for Twin Studies* **16**, 399-402, doi:10.1017/thg.2012.86 (2013).

28 Kremen, W. S. *et al.* Genes, environment, and time: the Vietnam Era Twin Study of Aging (VETSA). *Twin research and human genetics : the official journal of the International Society for Twin Studies* **9**, 1009-1022, doi:10.1375/183242706779462750 (2006).

29 Schoenborn, C. A. & Heyman, K. M. Health characteristics of adults aged 55 years and over: United States, 2004-2007. *National health statistics reports*, 1-31 (2009).

30 Winkler, T. W. *et al.* Quality control and conduct of genome-wide association meta-analyses. *Nature protocols* **9**, 1192-1212, doi:10.1038/nprot.2014.071 (2014).

31 Kanehisa, M., Sato, Y., Kawashima, M., Furumichi, M. & Tanabe, M. KEGG as a reference resource for gene and protein annotation. *Nucleic acids research* **44**, D457-462, doi:10.1093/nar/gkv1070 (2016).
