# Supplementary material for: Genetic correlations and genome-wide associations of cortical structure in general population samples of 22,824 adults
Source: Nat Commun. 2020 Sep 22;11:4796. doi: 10.1038/s41467-020-18367-y (PMC7508833; doi:10.1038/s41467-020-18367-y)
Supplement: Supplementary file 3 — Descriptions of Additional Supplementary Files [file 41467_2020_18367_MOESM3_ESM.pdf]

Descriptions of Additional Supplementary Files

Supplementary Data 1
Description: Genome-wide significant associations ($p_{Discovery} < 1.09 \times 10^{-9}$) of the global cortex.

Supplementary Data 2
Description: Genome-wide significant associations $_{(pDiscovery<1.09x10-9)}$ of regional cortical thickness.

Supplementary Data 3
Description: Genome-wide significant associations $_{(pDiscovery<1.09x10-9)}$ of regional cortical surface area.

Supplementary Data 4
Description: Genome-wide significant associations $_{(pDiscovery<1.09x10-9)}$ of regional cortical volume.

Supplementary Data 5
Description: Genomic control λ_{GC}, LDSR intercept and LDSR ratio.

Supplementary Data 6
Description: Genome-wide significant associations $_{(pDiscovery<1.09x10-9)}$ of surface area and volume in different cortical regions.

Supplementary Data 7
Description: Genome-wide significant associations $_{(pDiscovery<1.09x10-9)}$ within the same cortical region.

Supplementary Data 8
Description: Genome-wide significant associations ($p_{Discovery} < 1.09 \times 10^{-9}$) of cortical thickness, surface area and volume and corresponding UKBB p-values from Elliott, L.T. et al. 2018 and Zhao, B. et al. 2019.

Supplementary Data 9
Description: Polygenic risk score (PRS) prediction in 7800 out-of-sample individuals from the UK Biobank.

Supplementary Data 10
Description: GWAScatalog (as implemented in FUMA) results for lead SNPs ($P_{Discovery}<^1.09x^10^{-9}$) - cortical thickness.

Supplementary Data 11
Description: GWAScatalog (as implemented in FUMA) results for lead SNPs ($P_{Discovery}$<$1.09x10^{-9}$) - cortical surface area.

Supplementary Data 12
Description: GWAScatalog (as implemented in FUMA) results for lead SNPs $_{(PDiscovery<1.09x10-9)}$ - cortical volume.

Supplementary Data 13
Description: Annotation of genes mapped to independent lead SNPs - cortical thickness.

Supplementary Data 14
Description: Annotation of genes mapped to independent lead SNPs - cortical surface area.

Supplementary Data 15
Description: Annotation of genes mapped to independent lead SNPs - cortical volume.

Supplementary Data 16
Description: MAGMA gene-based analyses (as implemented in FUMA) – cortical thickness.

Supplementary Data 17
Description: MAGMA gene-based analyses (as implemented in FUMA) - cortical surface area.

Supplementary Data 18
Description: MAGMA gene-based analyses (as implemented in FUMA) - cortical volume.

Supplementary Data 19
Description: Association between lead SNPs of cortical thickness, surface area and volume $_{(pDiscovery<1.09x10-9)}$ and expression QTL in autopsied brains from the ROSMAP sample.

Supplementary Data 20
Description: MAGMA gene-set analyses (as implemented in FUMA).

Supplementary Data 21
Description: Heritability estimates.

Supplementary Data 22
Description: Partitioned heritability - functional annotation.

Supplementary Data 23
Description: Partitioned heritability - cell-type-specific annotation.

Supplementary Data 24
Description: Genetic correlation between cortical thickness, surface area and volume within regions.

Supplementary Data 25
Description: Genetic correlation of cortical thickness between regions.

Supplementary Data 26
Description: Genetic correlation of surface area between regions.

Supplementary Data 27
Description: Genetic correlation of volume between regions.

Supplementary Data 28
Description: Genetic correlation between cortical thickness and other GWAS phenotypes.

Supplementary Data 29
Description: Genetic correlation between cortical surface area and other GWAS phenotypes.

Supplementary Data 30
Description: Genetic correlation between cortical volume and other GWAS phenotypes.

Supplementary Data 31
Description: Description of genes associated with global and regional cortical thickness, surface area and volume.

Supplementary Data 32
Description: Population characteristics.

Supplementary Data 33
Description: Genotyping, quality control, imputation and association.

Supplementary Data 34
Description: Image acquisition and processing.

Supplementary Data 35
Description: Descriptive statistics of cortical thickness (mm).

Supplementary Data 36
Description: Descriptive statistics of cortical surface area (mm^2).

Supplementary Data 37
Description: Descriptive statistics of cortical volume (mm^3).
